# Direct Comparison of Clinical Characteristics, Outcomes, and Risk Prediction in Patients with COVID-19 and Controls—A Prospective Cohort Study

**DOI:** 10.3390/jcm10122672

**Published:** 2021-06-17

**Authors:** Maurin Lampart, Marco Rüegg, Andrea S. Jauslin, Noemi R. Simon, Núria Zellweger, Ceylan Eken, Sarah Tschudin-Sutter, Stefano Bassetti, Katharina M. Rentsch, Martin Siegemund, Roland Bingisser, Christian H. Nickel, Stefan Osswald, Gabriela M. Kuster, Raphael Twerenbold

**Affiliations:** 1 Department of Cardiology and Cardiovascular Research Institute Basel (CRIB), University Hospital Basel, University of Basel, Spitalstrasse 2, 4056 Basel, Switzerland; maurin.lampart@usb.ch (M.L.); ceylan.eken@usb.ch (C.E.); stefan.osswald@usb.ch (S.O.); gabriela.kuster@usb.ch (G.M.K.); 2 Emergency Department, University Hospital Basel, Petersgraben 2, 4031 Basel, Switzerland; marco.rueegg@usb.ch (M.R.); andrea.jauslin@usb.ch (A.S.J.); noemi.simon@usb.ch (N.R.S.); roland.bingisser@usb.ch (R.B.); Christian.Nickel@usb.ch (C.H.N.); 3 Intensive Care Unit, University Hospital Basel, Petersgraben 2, 4031 Basel, Switzerland; nuria.zellweger@usb.ch (N.Z.); martin.siegemund@usb.ch (M.S.); 4 Division of Infectious Diseases and Hospital Epidemiology, University Hospital Basel, Petersgraben 2, 4031 Basel, Switzerland; sarah.tschudin@usb.ch; 5 Division of Internal Medicine, University Hospital Basel, Petersgraben 2, 4031 Basel, Switzerland; stefano.bassetti@usb.ch; 6 Laboratory Medicine, University Hospital Basel, Petersgraben 2, 4031 Basel, Switzerland; katharina.rentsch@usb.ch; 7 Department of Clinical Research, University of Basel, Petersplatz 1, 4001 Basel, Switzerland; 8 University Center of Cardiovascular Science and Department of Cardiology, University Heart and Vascular Center Hamburg, Martinistraße 52, 20251 Hamburg, Germany

**Keywords:** COVID-19, SARS-CoV-2, characteristics, outcome, comparison, risk prediction, controls

## Abstract

Most studies investigating early risk predictors in coronavirus disease 19 (COVID-19) lacked comparison with controls. We aimed to assess and directly compare outcomes and risk predictors at time of emergency department (ED) presentation in COVID-19 and controls. Consecutive patients presenting to the ED with suspected COVID-19 were prospectively enrolled. COVID-19-patients were compared with (i) patients tested negative (overall controls) and (ii) patients tested negative, who had a respiratory infection (respiratory controls). Primary outcome was the composite of intensive care unit (ICU) admission and death at 30 days. Among 1081 consecutive cases, 191 (18%) were tested positive for severe acute respiratory syndrome coronavirus 2 (SARS-CoV-2) and 890 (82%) were tested negative (overall controls), of which 323 (30%) had a respiratory infection (respiratory controls). Incidence of the composite outcome was significantly higher in COVID-19 (23%) as compared with the overall control group (10%, adjusted-HR 2.45 (95%CI, 1.61–3.74), *p* < 0.001) or the respiratory control group (10%, adjusted-HR 2.93 (95%CI, 1.66–5.17), *p* < 0.001). Blood oxygen saturation, age, high-sensitivity troponin, c-reactive protein, and lactate dehydrogenase were identified as the strongest predictors of poor outcome available at time of ED presentation in COVID-19 with highly comparable prognostic utility in overall and respiratory controls. In conclusion, patients presenting to the ED with COVID-19 have a worse outcome than controls, even after adjustment for differences in baseline characteristics. Most predictors of poor outcome in COVID-19 were not restricted to COVID-19, but of comparable prognostic utility in controls and therefore generalizable to unselected patients with suspected COVID-19.

## 1. Introduction

Coronavirus disease 2019 (COVID-19), caused by the severe acute respiratory syndrome coronavirus 2 (SARS-CoV-2), is a global pandemic with a massive burden on healthcare systems worldwide. Early and reliable risk prediction at time of emergency department (ED) presentation is crucial to identify individuals at high risk of adverse outcome events and allocate limited health care resources. Multiple national and international medical associations have tried to define guidelines on triage criteria in COVID-19, which have led to controversial discussions including ethical aspects [1,2,3].

Early studies from China, Italy, and the USA analyzed characteristics and the predictive value of clinical parameters [4,5,6,7,8,9,10,11]. Various laboratory parameters such as high c-reactive protein (CRP), low albumin and leukocyte levels were found characteristic for COVID-19 [8], whereas higher age, obesity, hypertension, and high CRP concentrations were associated with poor outcome [10,12,13,14,15,16,17,18]. Overall, reported incidences of 30-day mortality, intensive care unit (ICU) admission, acute respiratory distress syndrome (ARDS), and intubation were high and varied widely between different regions and health care systems [4,6,9,10,11]. In addition, particularly during the early phase of the COVID-19 pandemic, data on outcomes and predictive value of clinical characteristics were mainly reported from heavily affected regions. Therefore, this data may not be generalizable to less affected regions such as Central Europe or Japan. Furthermore, most of the existing data was derived from retrospective registries enrolling exclusively patients with confirmed COVID-19 [19,20] or comparing hospitalized COVID-19 patients with influenza patients from the past three years [21,22,23]. To date, there is little literature that prospectively compares COVID-19 with an adequate control group of patients presenting within the same time period to the ED with diseases causing symptoms similar to COVID-19 (e.g., cough, fever, dyspnea [4,8]). There is a large overlap in symptoms and clinical features observed in COVID-19 and, for example, pneumonia, which complicates diagnosis and risk prediction in those patients. To assess the predictive value of clinical parameters and test whether they are COVID-19-specific or generalizable to unselected patients with similar symptoms of acute respiratory infection, a control group is mandatory.

Therefore, our aim was to assess clinical characteristics, outcomes, and the predictive value of clinical and laboratory parameters available at time of ED presentation in COVID-19 patients and to compare them with controls presenting with similar symptoms but no COVID-19 in a prospective setting. To support early clinical decision making, we further aimed to develop a simple risk prediction score combining the most predictive clinical parameters and to compare it with the already established CURB-65-Score for pneumonia [24].

## 2. Materials and Methods

### 2.1. Study Design, Population, and Inclusion Criteria

The prospective, observational, COronaVIrus surviVAl (COVIVA, ClinicalTrials.gov NCT04366765) cohort study included unselected patients aged 18 years and older presenting with clinically suspected or confirmed SARS-CoV-2 infection to the emergency department of the University Hospital Basel, Switzerland, during the first wave of COVID-19 pandemic between 23 March 2020 and 7 June 2020. All patients underwent nasopharyngeal SARS-CoV-2 polymerase chain reaction (PCR) swab tests. Patients were considered SARS-CoV-2 positive if one or multiple SARS-CoV-2 PCR swab tests performed at day of ED presentation or within 14 days prior to or post ED presentation were positive in combination with clinical signs and symptoms. The remainders with only negative SARS-CoV-2 swab test results were considered as controls. All participating patients or their legally authorized representatives consented by signing a local general consent form. This study was conducted according to the principles of the Declaration of Helsinki and approved by the local ethics committee (EKNZ identifier 2020-00566). The authors designed the study, gathered, and analyzed the data according to the STROBE guidelines (Table S1), vouched for the data and analysis, wrote the paper, and decided to submit it for publication [25].

### 2.2. Clinical Assessment

All patients underwent a thorough clinical assessment by the treating ED physician according to local standard operating procedures. Vital parameters including heart rate, blood pressure, oxygen saturation, and respiratory rate were assessed in every patient. For the interpretation of oxygen saturation, Basel is located 260 m above sea level.

### 2.3. Blood Sampling

Blood samples were routinely drawn in every patient (both COVID-19 and controls) at time of ED presentation. Besides routine laboratory parameters, high-sensitivity troponin T (hs-cTnT), N-terminal prohormone B-type natriuretic peptide (NT-proBNP), procalcitonin, and ferritin were measured for every patient as part of the local standard operating procedure for suspected COVID-19 patients. Timing and type of subsequent laboratory measurements during hospital stay were left to the discretion of the treating physicians and were not part of this study protocol.

### 2.4. Follow-Up

Thirty days after discharge, patients were contacted by telephone or in written form by research physicians or study nurses, and information about current health, hospitalizations, and adverse outcome events was collected using a predefined set of questions and item-checklists. Records of hospitals and primary care physicians as well as national death registries were screened for additional information, if applicable.

### 2.5. Outcomes

The primary outcome measure aimed to reflect disease severity and was defined as the composite of ICU admission or all-cause death at 30 days. Secondary outcomes included death at 30 days, ICU admission, patient management, length of hospital stay as well as incidence of intubation, hemodynamic support, ARDS during the index hospitalization and rehospitalization for respiratory reasons at 30 days.

### 2.6. Adjudication of Final Diagnosis

To determine the final diagnosis that led to the index ED presentation and the clinical suspicion of COVID-19, five trained physicians reviewed all medical data available including 30 days post-discharge follow-up information. They chose from a predefined list of diagnoses what best fit each patient. Each adjudication was primarily assigned by one physician per patient only. However, all uncertain cases were discussed collectively within the adjudicating team and final decision was made in the consensus by majority vote. Predefined main categories included but were not limited to COVID-19, non-SARS-CoV-2 infections (e.g., other respiratory, gastrointestinal, urogenital), cardiovascular disease (acute coronary syndrome, rhythm disorder, congestive heart failure, pulmonary embolism), other pulmonary non-infectious diseases (e.g., lung tumor, asthma, chronic obstructive pulmonary disease), and neurologic diseases (e.g., stroke, seizure).

### 2.7. Statistical Analysis

Data are expressed as medians and interquartile range (IQR) for continuous variables, and as numbers and percentages (%) for categorical variables. All variables were compared by Mann Whitney U test for continuous variables and Pearson chi-square or Fisher’s exact test for categorical variables, as appropriate. In this analysis, COVID-19 patients were compared with two control groups: First, with unselected, SARS-CoV-2 negative patients (overall controls) and second with the subgroup of patients with acute respiratory infections but no COVID-19 (respiratory controls, e.g., viral infection of the upper airways, bacterial pneumonia). The primary composite outcome was plotted in Kaplan-Meier curves, and the log-rank test was used to assess differences between groups. A Cox proportion hazard model was used to assess the prognostic value of clinical parameters in COVID-19 compared with the controls in a univariable approach as well as adjusted for co-variables considered relevant for the clinical course in COVID-19 based on existing literature and clinical judgement (i.e., cardiac disease, pneumopathy, overweight (BMI > 25 kg/m^2^), diabetes, active smoking, CRP, and blood oxygen saturation at time of ED presentation) in a multivariable approach. For the derivation of a multivariable risk score, best performing demographic parameters, comorbidities, symptoms, vital signs, and laboratory parameters in univariable analysis were considered as candidates in multivariable models. Collinearity was assessed by the variance inflation factor, accepting levels <2 for inclusion in multivariable analysis. In case of collinearity between variables, the selection of the variable entering the multivariable model was based on availability and relevance in clinical practice as well as performance in the univariable model. Given the limited sample size, a maximal number of ten co-variables were included in the initial multivariable Cox proportion hazard model. Independent predictors were then identified using a stepwise-backward selection process on the unaltered variables. We used the Youden-index and visual classifications to define optimal cut-off values and weighted the impact of the predictors based on coefficients obtained by binary logistic regressions. For missing values, multiple imputation was applied for variables with maximally 15% of missing data (Table S2). As predictor measures for the multiple imputation, we used seven additional variables (age, sex, coronary artery disease, diabetes, creatinine, leukocytes, and the primary composite outcome measure). Rubin’s rule was used to combine the results [26]. To assess the discriminative performance of the newly derived score for the primary composite outcome measure as well as 30-day mortality, we calculated the area under the receiver operating characteristic curve (AUROC) for all the variables embedded in the score separately as well as for the score itself. A value of 0.5 indicates no predictive ability, a value of 0.8 is considered good, and 1.0 is perfect. We plotted model calibration curves to examine agreement between predicted and observed risk across deciles of event risk to determine the presence of over- or underprediction. The newly derived score was compared with the established CURB-65-Score (Confusion, Urea, Respiratory rate, Blood pressure, age ≥65) for pneumonia using AUROC and categorized net reclassification improvement (NRI) [27,28]. The CURB-65-Score was designed to estimate the risk of mortality for community acquired pneumonia (CAP) and is still recommended for predicting the outcome of CAP [24,29]. For NRI calculation, the CURB-65-Score was categorized in low (0–1 points), intermediate (2 points), and high risk (3–5 points) as suggested by Ebell [30]. The two scores were compared in COVID-19, respiratory controls, and in COVID-19 and respiratory controls combined, because this combination matches the group of patients the most accurately, that would qualify for a risk prediction with the CURB-65-Score at the ED. For the direct comparison of the primary composite endpoint in COVID-19 and controls, *p*-values of less than 0.05 were considered significant. No correction for multiple testing was applied. Statistical analyses were performed using IBM SPSS Statistics for Windows, version 27.0 (IBM Corp., Armonk, NY, USA) and MedCalc for Windows, version 19.8 (MedCalc Software, Ostend, Belgium).

## 3. Results

### 3.1. Baseline Characteristics in COVID-19 and Controls

Overall, 1202 cases of patients presenting with symptoms suggesting COVID-19 were screened and 1086 were enrolled in this study from 23 March 2020 to 7 June 2020. Follow-up 30 days after discharge was completed in 1081 cases. COVID-19 was confirmed in 191 cases (18%). Among the 890 cases (82%) without COVID-19 (overall controls), 323 cases (30%) were diagnosed with an acute respiratory infection other than COVID-19 (respiratory controls, Figure S1).

The demographic and clinical characteristics of COVID-19 patients and controls are shown in Table 1. Median age was 59 years (IQR, 42–73) and 469 (43%) were females with no significant differences between the three groups. Prevalence of most comorbidities and cardiovascular risk factors did not differ between the three groups, except for cardiac disease, atrial fibrillation, pneumopathy, overweight, and smoking, which were less frequent in COVID-19. Patients with COVID-19 presented to the ED later after symptom onset (median 7 days, (IQR, 3–11) as compared with overall controls (median 3 days, (IQR, 2–8)) or respiratory controls (median 4 days, (IQR, 2–9)). The most common symptoms at ED presentation were cough and dyspnea. Cough was prevalent in 66% in COVID-19 versus 52% in overall controls (*p* = 0.001) and 75% in respiratory controls (*p* = 0.03). Dyspnea was reported in 42% in COVID-19 versus 49% in overall controls (*p* = 0.088) and 57% in respiratory controls (*p* = 0.001). Vital signs at time of ED presentation were comparable in all three groups. In contrast, notable differences in the laboratory parameters were observed between the three groups: In COVID-19, leukocyte and lymphocyte levels were significantly lower, while CRP, ferritin, and lactate dehydrogenase (LDH) levels were significantly higher than in the overall and respiratory controls.

### 3.2. Patient Management and Outcome in COVID-19 and Controls

In COVID-19, 60% of patients were managed as inpatients compared with 50% in the overall controls (*p* = 0.014) and 43% in the respiratory controls (*p* < 0.001). Among inpatients, median length of stay was 7 days (IQR, 4–13) in COVID-19 versus 6 days (IQR, 3–10) in both controls (*p* = 0.013 and *p* = 0.003, respectively). Baseline characteristics of inpatients only can be found in Table S3 in the Supplementary Materials. Among inpatients, COVID-19 was associated with younger age, fewer comorbidities, and higher acute phase proteins as compared to hospitalized overall controls and respiratory controls.

**Figure 1:**
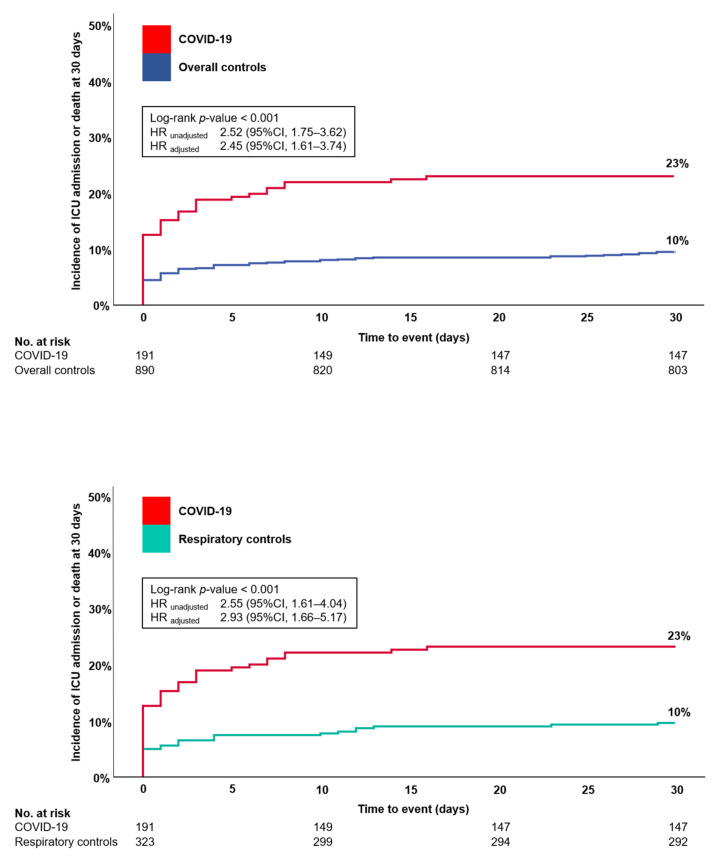
Event curve for ICU admission and death at 30 days in COVID-19 and controls. First panel shows incidence of ICU admission and death at 30 days for COVID-19 vs. overall controls. Second panel shows incidence of ICU admission and death at 30 days for COVID-19 vs. respiratory controls, adjustments were made for cardiac disease, pneumopathy, overweight, diabetes, active smoking, CRP, and blood oxygen saturation; ICU = intensive care unit, COVID-19 = coronavirus disease 2019, HR = hazard ratio, CI = confidence interval, CRP = c-reactive protein.

Incidence of the primary composite outcome, consisting of ICU admission and death at 30 days, was higher in COVID-19 (44/191, 23%) than in the overall controls (87/890, 10%, log-rank *p*-value < 0.001) or the respiratory controls (31/323, 10%, log-rank *p*-value < 0.001, Table 2, Figure 1). In COX proportional hazard analysis, COVID-19 was associated with an increased risk of the primary composite outcome versus overall controls (unadjusted HR 2.52 (95%CI, 1.75–3.62), *p* < 0.001) and versus respiratory controls (unadjusted HR 2.55 (95%CI, 1.61–4.04), *p* < 0.001), respectively, which persisted even after adjustment for cardiac disease, pneumopathy, overweight, diabetes, active smoking, CRP and blood oxygen saturation at time of ED presentation (for comparison with overall controls, adjusted HR 2.45 (95%CI, 1.61–3.74), *p* < 0.001; for comparison with respiratory controls, adjusted HR 2.93 (95%CI, 1.66–5.17), *p* < 0.001).

In COVID-19, incidence of ICU admission was 21%, 14% developed a documented ARDS, 15% needed hemodynamic support, and 16% needed intubation. In overall controls only 7% were admitted to the ICU, 1% developed ARDS, 3% needed hemodynamic support, and 3% needed intubation. In respiratory controls, only 7% were admitted to the ICU, 1% developed ARDS, 4% needed hemodynamic support, and 5% needed intubation. In COVID-19, 30-day mortality was 7% compared to 4% in both controls (*p*-values not significant, Figure S2).

### 3.3. Direct Comparison of Clinical Characteristics between Outcomes in COVID-19 and Controls

Table 3 displays the baseline characteristics with respect to the incidence of the primary composite outcome at 30 days in COVID-19 and controls. Higher age was associated with a poor outcome in all three groups. In contrast, overweight was identified as a significant risk predictor only in COVID-19 but not in controls with a two-fold higher prevalence of overweight in patients with poor outcome than in event-free patients (61% versus 32%, *p* < 0.001). Patients with a poor outcome presented to the ED sooner after symptom onset in all three groups, while prevalence of cough and dyspnea did not differ between events and non-events in all three groups. Median respiratory rates were higher and blood oxygen saturation was lower in patients with poor outcome in all three groups. Multiple laboratory parameters showed substantial differences between events and non-events in all three groups including leukocytes, lymphocytes, CRP, ferritin, LDH, hs-cTnT, and NT-proBNP. E.g., in COVID-19, median CRP levels were 15.6 mg/dL (IQR 1.6–46.7) in event-free survivors as compared to 112.6 mg/dL (IQR 47.6–162.7) in patients with the primary composite outcome (*p* < 0.001).

### 3.4. Predictive Value of Clinical Parameters in COVID-19 and Controls

**Figure 2:**
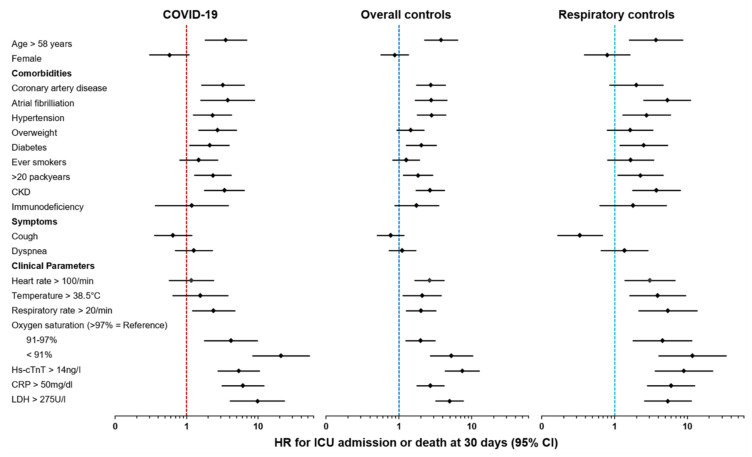
Forest plots for HR of clinical parameters for ICU admission and death at 30 days in COVID-19, overall controls, and respiratory controls. X-axis shows HR in logarithmic scaling. Higher HR suggests higher association with poor outcome; COVID-19 = coronavirus disease 2019, CKD = chronic kidney disease, hs-cTnT = high-sensitivity troponin T, CRP = c-reactive protein, LDH = lactate dehydrogenase, HR = hazard ratio, ICU = intensive care unit, CI = confidence interval.

The predictive value of a wide range of clinical parameters in COVID-19 as well as in overall controls and respiratory groups, as assessed using univariable COX proportional hazard analysis for the primary composite outcome, is displayed in Table 4 and Figure 2. Of note, the predictive value of most clinical variables was highly comparable between COVID-19 and both controls. E.g., higher age was associated with an increased risk of the primary outcome in COVID-19 (HR per decade 1.42 (95%CI, 1.18–1.71)), but also in overall controls (HR per decade 1.35 (95%CI, 1.20–1.52)), and respiratory controls (HR per decade 1.35 (95%CI, 1.11–1.66)).

In multivariable COX proportional hazard analysis, high levels of CRP, hs-cTnT, and LDH as well as low blood oxygen saturation, and older age were identified as the strongest predictors of poor outcome in COVID-19. Based on these five widely available variables, we developed the COLT-58-Score (CRP, Oxygen Saturation, LDH, Troponin, Age > 58). One point was assigned for CRP > 50 mg/dL, LDH > 275 U/L, hs-cTnT > 14 ng/L, and age > 58 years, each. As blood oxygen saturation was the strongest predictor in the multivariable Cox proportional hazard analysis, it was categorized into three instead of two groups (Figure S3). One point was assigned for oxygen saturations at time of ED presentation ranging between 91–96% and two points for oxygen saturations < 91%, accounting for a summed overall score ranging from 0–6 points (Table 5).

**Figure 3:**
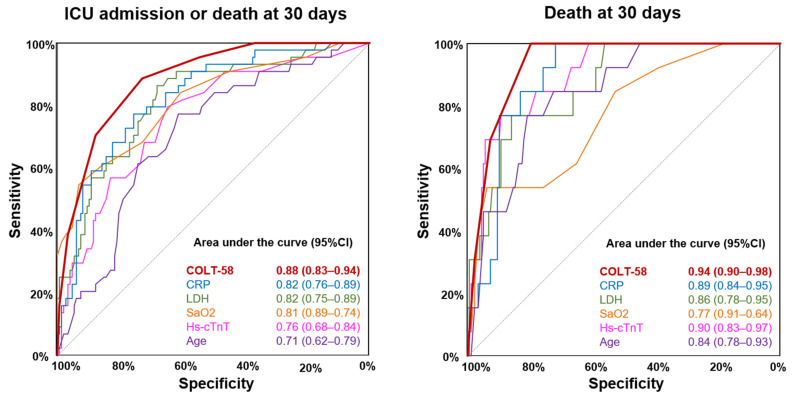
Predictive performance of the COLT-58-Score and its components in COVID-19. Left panel shows the AUROC for the primary composite outcome consisting of ICU admission and death at 30 days for the COLT-58-Score, CRP, LDH, SaO2, hs-cTnT, and age in COVID-19; Right panel shows the AUROC for death at 30 days for the COLT-58-Score, CRP, LDH, SaO2, hs-cTnT, and age; COVID-19 = coronavirus disease 2019, AUROC = area under the receiver operating characteristic curve, ICU = intensive care unit, CI = confidence interval, CRP = c-reactive protein, LDH = lactate dehydrogenase, SaO2 = blood oxygen saturation, hs-cTnT = high-sensitivity troponin T.

The COLT-58-Score resulted in high discriminative accuracy for the prediction of the primary composite outcome in COVID-19 (AUROC 0.88 (95%CI, 0.83–0.94)), which was superior to all its single components and even higher for the prediction of 30-day mortality (AUROC 0.94 (95%CI, 0.90–0.98), Figure 3). In overall controls, the COLT-58-Score also resulted in high discriminative accuracy for the primary outcome (AUROC 0.79 (95%CI, 0.74–0.84)) and for 30-day mortality (AUROC 0.85 (95%CI, 0.80–0.90)), but lower than in COVID-19. Similarly, in respiratory controls, the COLT-58-Score resulted in high discriminative accuracy for the primary outcome (AUROC 0.85 (95%CI, 0.79–0.91)) and for 30-day mortality (AUROC 0.88 (95%CI, 0.80–0.96)), but lower than in COVID-19 (Figure S4).

**Figure 4:**
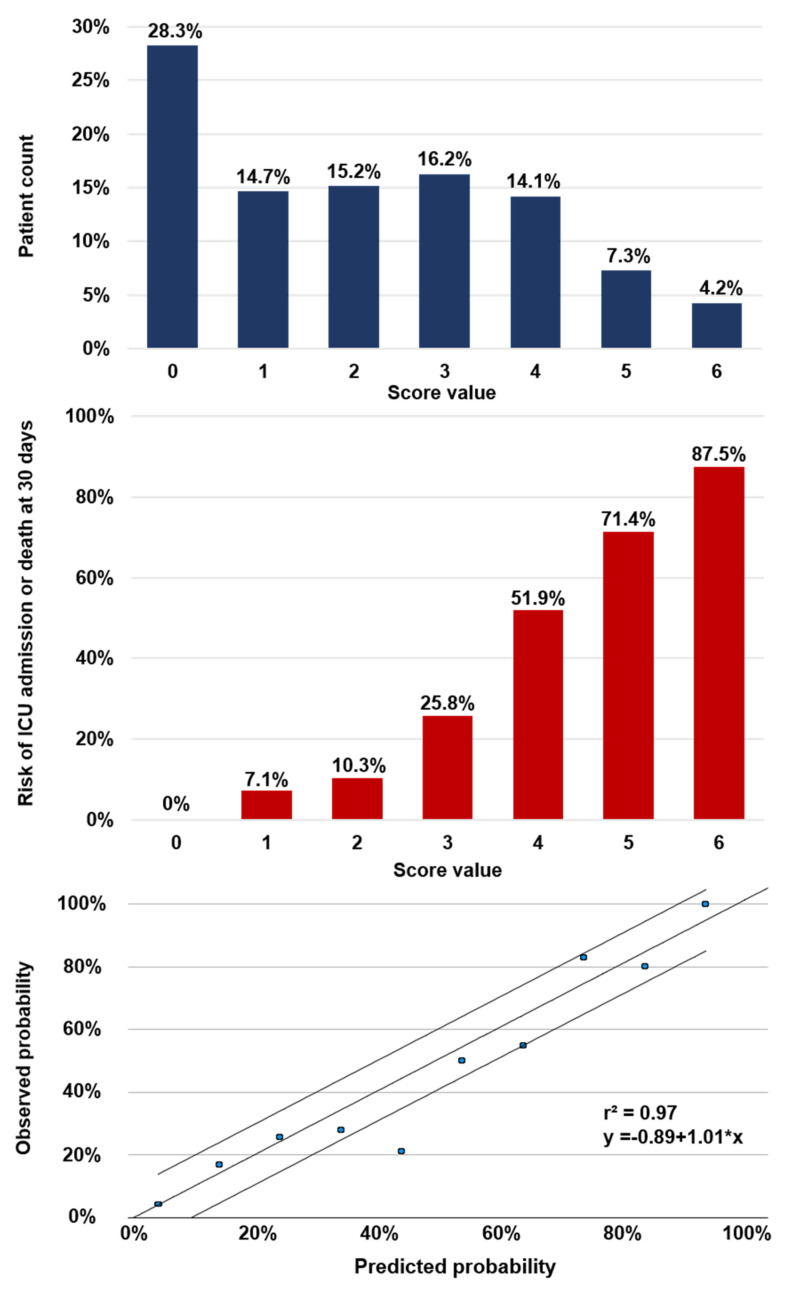
Characteristics of the COLT-58-Score in COVID-19. Upper panel shows distribution of patients for the respective score value. Middle panel shows the incidence of the primary composite outcome of ICU admission and death at 30 days for the respective score value. Lower panel shows the plotted calibration curve, x-axis shows the predicted probability, y-axis shows the observed probability of the primary composite outcome of ICU admission and death at 30 days; ICU = intensive care unit.

Figure 4 shows the frequency distribution of the COLT-58-Score and the associated risk of the primary composite outcome at 30 days for each score value in COVID-19. E.g., COVID-19-patients with a summed score of 0 had a 0% (95%CI, 0–6.6%) risk of poor outcome, whereas patients with a maximum summed score of 6 had a risk of 87.5% (95%CI, 52.9–97.8%). To assess the level of calibration, high correlation between estimated and observed risk was documented (r^2^ = 0.97, an intercept of –0.89 and a slope of 1.01). Diagnostic performance measures of multiple cut-off criteria for the COLT-58-Score regarding rule-out and rule-in are listed in Table 6.

### 3.5. Comparison of the COLT-58-Score with the CURB-65-Score

To further assess the clinical utility of the COLT-58-Score, we directly compared it with the well-established CURB-65-Score in COVID-19, respiratory controls, and patients with any respiratory infection (COVID-19 plus respiratory controls) using AUROC and the NRI (Figure S5, Table S4). The COLT-58-Score was categorized in low (0–2 points), intermediate (3–4 points), and high risk (5–6 points) for the calculation of the NRI. For prediction of the primary composite outcome in COVID-19, the COLT-58-Score showed significantly higher discriminative accuracy than the CURB-65-Score (AUROC 0.88 (95%CI, 0.83–0.94) versus AUROC 0.77 (95%CI, 0.69–0.86), *p* < 0.001) and significantly improved reclassification (NRI 30.04%, *p* = 0.013). Similarly, in patients with any respiratory infection, the COLT-58-Score showed significantly higher discriminative accuracy than the CURB-65-Score (AUROC 0.87 (95%CI, 0.83–0.91)) versus AUROC 0.77 (95%CI, 0.71–0.83), *p* < 0.001) and significantly improved reclassification (NRI 26.92%, *p* = 0.003) for the prediction of the primary composite outcome. In respiratory controls, the CURB-65-Score and the COLT-58-Score showed comparable classification with no significant difference in AUROC or the NRI. Similarly, no significant differences between the two scores were observed for the prediction of 30-day mortality in all three groups, however the COLT-58-Score showed numerically higher discriminative accuracy in AUROC.

## 4. Discussion

### 4.1. Findings

In this observational single-center cohort study of patients presenting with suspected SARS-CoV-2 infection to the ED of the University Hospital in Basel, Switzerland, we explored and directly compared the characteristics, outcomes, and predictive value of a wide range of clinical parameters in COVID-19 and control patients. We report five major findings.

First, whereas symptoms and vital signs at ED presentation were largely comparable between COVID-19 and controls, numerous laboratory parameters differed significantly. These included lower leukocyte and lymphocyte counts in COVID-19 compared to controls. Low leukocytes in COVID-19 have been reported already early during the COVID-19 pandemic [4,8]. Our study adds to this observation by demonstrating, that leukocyte and lymphocyte counts at time of ED presentation are lower in COVID-19 than in diseases causing similar symptoms, including patients with acute respiratory infections other than COVID-19. Of note, these findings are not necessarily linked with COVID-19-specific mechanisms but may also be partly explained by the later presentation of COVID-19 patients after symptom onset. In contrast, CRP and ferritin were significantly higher in COVID-19 than in controls, suggesting higher inflammatory activity in COVID-19 at time of ED presentation in.

Second, COVID-19 was associated with worse outcome compared to patients presenting at the same time with similar symptoms but without COVID-19. The risk of the primary composite outcome of ICU admission and death at 30 days was twice as high in COVID-19 as in controls, even after adjustment for comorbidities and differences in baseline characteristics. Similarly, 30-day mortality alone was numerically higher in COVID-19 than in controls, although not reaching level of significance. In parallel, the risk of intubation was increased five-fold in COVID-19 compared to controls, and the risk of ARDS was increased more than ten-fold. In early studies during the COVID-19 pandemic, incidences of ICU admission, 30-day mortality, and need for intubation varied widely and were numerically higher compared to our results [4,6,9,10]. However, direct comparison is difficult since health care systems in early studies were often at or beyond the absolute limit of their capacities. More recent studies from Central Europe and Japan with a health care system still standing showed comparable results to our study regarding rates of ICU admission, 30-day mortality, and intubation in COVID-19 [19,20,21,22,23].

Third, a multitude of clinical parameters available upon ED presentation were associated with poor outcome in both COVID-19 and controls. Of note, however, their predictive value did not systemically differ between COVID-19 and controls. Specifically, older age, history of cardiac disease, hypertension, diabetes, CKD, and long-standing smoking were significantly more prevalent in patients with poor outcome in COVID-19 and controls. In contrast, overweight was more prevalent in COVID-19 than in controls and association of overweight with poor outcome could only be observed in COVID-19. Numerous papers have already demonstrated the negative prognostic role of overweight and obesity in COVID-19 [12,13,18,31]. However, it was unclear whether this phenomenon is generalizable or to some extent COVID-19 specific, as suggested by our data. No difference in outcome was observed for presence of cough or dyspnea. These findings underline the limited predictive value of clinical symptoms at time of ED presentation, which can be misleading and therefore should not be overestimated when evaluating risk of poor outcome. Increased heart rate was only associated with poor outcome in controls but not in COVID-19. This may be explained by reported relative bradycardia in some COVID-19 patients [32,33]. Most laboratory parameters were associated with poor outcome in all groups. Most impressively, inflammation markers such as CRP and ferritin showed a high association with poor outcome in all groups, but most pronounced in COVID-19, which confirms and corroborates results from earlier studies [4,10,14,15]. Levels of leukocytes were systematically higher in patients with poor outcome in all three groups. However, leukocyte levels in COVID-19 patients with poor outcome were lower than leukocyte levels in control patients with favorable outcome. This adds to the theory that SARS-CoV-2 infests leukocytes and thereby leads to a relative leukopenia [4,8].

Fourth, CRP, blood oxygen saturation, LDH, hs-cTnT, and age were strong predictors of poor outcome in COVID-19. Combined in a novel, simple risk score (COLT-58-Score), these five measures showed high utility to predict the primary composite outcome and death at 30 days in COVID-19. Most of these parameters have already been identified as predictors of poor outcome in a single marker approach [16,17,34], but they have never been combined to one risk score to predict the outcome in COVID-19. Of note, besides patients with confirmed COVID-19, in which the score was trained, the COLT-58-Score still performed well in respiratory controls and in the mixed group of patients with any respiratory infection regardless of the presence or absence of COVID-19.

Finally, the COLT-58-Score showed a significantly better discrimination and reclassification than the established CURB-65-Score in predicting the primary composite outcome in COVID-19 and in patients with any respiratory infection. Regarding the prediction of 30-day mortality only, the prognostic accuracy of the COLT-58-Score was even higher than for the primary outcome and comparable to the CURB-65-Score in all three subgroups. These findings suggest the potential clinical utility of the COLT-58-Score in patients with suspected COVID-19 and invites to validate this score in different cohorts.

### 4.2. Strength and Limitations

Our study has several strengths and limitations. The first strength is its prospective design in unselected ED patients. To our knowledge, there is a systematic lack of prospective cohort studies assessing clinical characteristics and outcomes of COVID-19. This comes with the advantages of minimizing a potential recall bias and more complete data collection. The second strength is the presence of large, representative control groups. These allow the direct comparison of clinical characteristics and outcomes observed in COVID-19 with patients presenting with similar symptoms but no COVID-19. The presence of a control group is mandatory to compare the predictive value of clinical parameters and to test whether they are COVID-19-specific or generalizable to unselected patients presenting with symptoms suggestive of COVID-19. The third strength is the routine measurement of a range of laboratory parameters at time of ED presentation including hs-cTnT, NT-proBNP, and ferritin in all patients.

There are, however, also several limitations. First, only 191 patients were tested SARS-CoV-2 positive and only 44 events of the primary composite endpoint were recorded. Due to the rather small sample size and event numbers, this study has limited power for extensive multivariable analysis. This must be particularly considered when interpreting the findings of the multivariable COLT-58-Score. However, given its prospective design with integrated control groups, the observed results still may add valuable information to the literature. Second, despite our efforts to minimize the error of misclassification by carefully analyzing available SARS-CoV-2 PCR test results from 14 days prior and after the initial ED visit, there is still the possibility of some false negatives in the respective control groups. Third, our study was performed in one Swiss tertiary hospital with a rather low prevalence of COVID-19, which may reduce generalizability of our findings to different settings with substantially higher prevalence. However, as the observed event rates are comparable with data from other Central European countries, generalizability of our findings to such countries can be assumed [19,21,22]. Similarly, distribution of risk predictors may differ substantially between various geographic regions and health care systems, which may also impact their prognostic and clinical relevance. Our dataset reflects a Central European setting with a compensated health care setting during the COVID-19 pandemic. Fourth, due to the lack of internal and external validation of the COLT-58-Score, further validation in future studies is absolutely mandatory prior to its clinical implementation. Fifth, this study contains numerous comparisons with no a-priori adjustment for multiple testing. Accordingly, *p*-values must be interpreted with caution. Last, between the start of our study and today, the proposed treatment of COVID-19 has changed [35,36,37], potentially impacting outcomes in COVID-19.

## 5. Conclusions

In this prospective Central European cohort study, patients presenting to the ED with COVID-19 have a worse outcome than controls, even after adjustment for differences in baseline characteristics. In general, most predictors of poor outcome in COVID-19 were not restricted to COVID-19, but generalizable to controls.

## Figures and Tables

**Table 1:** Baseline characteristics in COVID-19 and controls.

Measures	COVID-19	Overall Controls	*p*-Value ^1^	Respiratory Controls	*p*-Value ^2^
	*n* = 191	*n* = 890		*n* = 323	
Demographics					
Age—years	57 (44–69)	59 (41–74)	0.365	58 (42–71)	0.999
Female	84 (44)	385 (43)	0.855	142 (44)	0.997
Comorbidities–no (%)					
Cardiac disease	38 (20)	261 (29)	0.008	92 (28)	0.030
Valvular cardiopathy	8 (4)	54 (6)	0.311	16 (5)	0.691
Coronary artery disease	21 (11)	131 (15)	0.179	43 (13)	0.442
Prior myocardial infarction	9 (5)	70 (8)	0.129	22 (7)	0.334
Atrial fibrillation	9 (5)	91 (10)	0.017	33 (10)	0.028
Hypertension	81 (42)	367 (41)	0.765	142 (44)	0.731
Overweight	74 (39)	278 (31)	0.045	91 (28)	0.013
Diabetes	36 (19)	145 (16)	0.391	53 (16)	0.480
Ever smoker	58 (30)	361 (41)	0.009	159 (49)	<0.001
-Active smoker	20 (10)	209 (23)	<0.001	98 (30)	<0.001
-Packyears > 20	18 (9)	164 (18)	0.003	80 (25)	<0.001
Pneumopathy	37 (19)	267 (30)	0.003	127 (39)	<0.001
-Asthma	25 (13)	112 (13)	0.849	54 (17)	0.270
-COPD	9 (5)	110 (12)	0.002	58 (18)	<0.001
Hepatopathy	14 (7)	104 (12)	0.080	37 (11)	0.131
CKD	26 (14)	145 (16)	0.357	39 (12)	0.612
Stroke	10 (5)	70 (8)	0.208	19 (6)	0.759
Cancer	17 (9)	93 (10)	0.521	30 (9)	0.883
Immunodeficiency	11 (6)	56 (6)	0.782	25 (8)	0.395
Symptoms at ED					
Symptom duration before ED—days	7 (3–11)	3 (2–8)	<0.001	4 (2–9)	0.002
Cough	126 (66)	465 (52)	0.001	242 (75)	0.030
Dyspnea	81 (42)	438 (49)	0.088	185 (57)	0.001
Vital signs at ED					
Systolic BP—mmHg	135 (122–148.5)	137 (121–156)	0.103	139 (122–155)	0.041
Diastolic BP—mmHg	82 (71–90)	81 (72–90)	0.758	82 (73–89)	0.988
Heart rate—/min	89 (80–103)	88 (75–103)	0.252	90 (76–104)	0.967
Blood oxygen saturation—%	97 (94–98)	97 (95–98)	0.009	97 (95–98)	0.274
Respiratory rate—/min	20 (16–24)	18 (16–23)	0.034	19 (16–23)	0.290
Temperature—°C	37.1 (36.8–38)	37.0 (36.5–37.7)	0.001	37.0 (36.6–37.5)	0.009
Laboratory parameters at ED					
Leukocytes—G/L	6.27 (4.95–8.34)	8.82 (6.82–11.70)	<0.001	9.08 (7.12–11.69)	<0.001
Lymphocytes—%	19.15 (11.85–26.85)	17.15 (9.80–26.60)	0.072	18.35 (9.45–26.85)	0.246
Lymphocytes absolute—G/L	1.07 (0.72–1.57)	1.47 (0.90–2.08)	<0.001	1.60 (1.00–2.19)	<0.001
Thrombocytes—G/L	218 (177–277)	240 (196–291)	0.004	249 (205–294)	<0.001
CRP—mg/dL	28.9 (2.6–73.4)	7.6 (1.2–47.6)	<0.001	9.0 (1.4–48.5)	0.001
Ferritin—µg/L	387 (164–823)	163 (85–329)	<0.001	162 (85–296)	<0.001
eGFR—mL/min/1.73 m²	93 (68–103)	89 (68–106)	0.708	92 (72–109)	0.109
Sodium—mmol/L	137 (134–140)	139 (136–141)	0.001	138 (136–141)	0.001
Potassium—mmol/L	3.9 (3.7–4.2)	4 (3.8–4.3)	0.027	4 (3.7–4.3)	0.119
LDH—U/L	254 (201–352)	215 (186–259)	<0.001	209 (185–254)	<0.001
ASAT—U/L	32 (23–45)	26 (21–33)	<0.001	26 (21–33)	<0.001
Albumin—g/L	34 (30–38)	37 (32–40)	<0.001	37 (32–40)	<0.001
Hs-cTnT—ng/L	7 (4–14)	9 (4–22)	0.033	7 (4–17)	0.573
NT-proBNP—pg/mL	77 (49–242)	115 (49–462)	0.019	97 (49–272)	0.359

^1^ *p*-value for comparison of COVID-19 with overall controls; ^2^ *p*-value for comparison of COVID-19 with respiratory controls. Continuous variables were compared using the Mann-Whitney-U test, and categorical variables using the Pearson χ2 test or Fisher’s exact test, as appropriate; missing variables are listed in Supplementary Table S2. Values are numbers (percentages) or median (interquartile range); COVID-19 = coronavirus disease 2019, COPD = chronic obstructive pulmonary disease, CKD = chronic kidney disease, ED = emergency department, BP = blood pressure, CRP = c-reactive protein, eGFR = estimated glomerular filtration rate, LDH = lactate dehydrogenase, ASAT = aspartate aminotransferase, hs-cTnT = high-sensitivity troponin T, NT-proBNP = N-terminal prohormone B-type natriuretic peptide.

**Table 2:** Patient management and outcomes in COVID-19 and controls.

Measures	COVID-19	Overall Controls	*p-*Value ^1^	Respiratory Controls	*p-*Value ^2^
	*n* = 191	*n* = 890		*n* = 323	
Patient management—no (%)					
Outpatient	77 (40)	446 (50)	0.014	185 (57)	<0.001
Inpatient	114 (60)	444 (50)		138 (43)	
Length of hospital stay—days					
-Overall	4 (0–9)	0 (0–6)	<0.001	0 (0–5)	<0.001
-Inpatients	7 (4–13)	6 (3–10)	0.013	6 (3–10)	0.003
Clinical course and outcomes					
ICU admission or death at 30 days	44 (23)	87 (10)	<0.001	31 (10)	<0.001
Death at 30 days	13 (7)	34 (4)	0.063	13 (4)	0.167
ICU admission	40 (21)	63 (7)	<0.001	23 (7)	<0.001
-Days at ICU	9 (4–16.5)	2 (1–4)	<0.001	3 (1.5–8.5)	<0.001
Intubation	30 (16)	23 (3)	<0.001	15 (5)	<0.001
-Days intubated	9 (6–12)	2 (1–7)	<0.001	3 (1–8)	<0.001
Hemodynamic support	28 (15)	26 (3)	<0.001	14 (4)	<0.001
ARDS	26 (14)	6 (1)	<0.001	4 (1)	<0.001
Rehospitalisation for respiratory reasons at 30 days	4 (2)	10 (1)	0.278	3 (1)	0.268

^1^ *p*-value for comparison of COVID-19 with overall controls; ^2^ *p*-value for comparison of COVID-19 with respiratory controls; Mann-Whitney-U test was used to compare continuous variables, Pearson χ2 test to compare patient management, and log-rank test to compare incidence of events at 30 days. Values are numbers (percentages) or median (interquartile range); COVID-19 = coronavirus disease 2019, ICU = intensive care unit, ARDS = acute respiratory distress syndrome.

**Table 3:** Baseline characteristics according to the primary composite outcome of admission to intensive care or death at 30 days in COVID-19 and controls.

Measures	COVID-19		*p*-Value ^1^	Overall Controls		*p*-Value ^1^	Respiratory Controls		*p*-Value ^1^
	*n* = 191			*n* = 890			*n* = 323		
	Composite Outcome			Composite Outcome			Composite Outcome		
	yes	no		yes	no		yes	no	
	*n* = 44	*n* = 147		*n* = 87	*n* = 803		*n* = 31	*n* = 292	
Demographics									
Age—years	66 (58–74)	54 (41–64)	<0.001	71 (60–79)	57 (39–73)	<0.001	70 (60–77)	56 (40–69.5)	0.001
Female	14 (32)	70 (48)	0.064	35 (40)	350 (44)	0.548	12 (39)	130 (45)	0.535
Comorbidities—no (%)									
Cardiac disease	19 (43)	19 (13)	<0.001	53 (61)	208 (26)	<0.001	21 (68)	71 (24)	<0.001
Valvular cardiopathy	4 (9)	4 (3)	0.064	11 (13)	43 (5)	0.007	3 (10)	13 (4)	0.202
Coronary artery disease	11 (25)	10 (7)	0.001	27 (31)	104 (13)	<0.001	7 (23)	36 (12)	0.11
Prior myocardial infarction	4 (9)	5 (3)	0.118	20 (23)	50 (6)	<0.001	4 (13)	18 (6)	0.157
Atrial fibrillation	6 (14)	3 (2)	0.001	20 (23)	71 (9)	<0.001	11 (35)	22 (8)	<0.001
Hypertension	27 (61)	54 (37)	0.004	57 (66)	310 (39)	<0.001	21 (68)	121 (41)	0.005
Overweight	27 (61)	47 (32)	<0.001	34 (39)	244 (30)	0.096	12 (39)	79 (27)	0.17
Diabetes	14 (32)	22 (15)	0.012	24 (28)	121 (15)	0.003	10 (32)	43 (15)	0.012
Ever smoker	17 (39)	41 (28)	0.174	40 (46)	321 (40)	0.279	19 (61)	140 (48)	0.158
-Active smoker	2 (5)	18 (12)	0.143	24 (28)	185 (23)	0.342	13 (42)	85 (29)	0.14
-Packyears > 20	8 (18)	10 (7)	0.023	25 (29)	139 (17)	0.009	13 (42)	67 (23)	0.02
Pneumopathy	9 (20)	28 (19)	0.836	27 (31)	240 (30)	0.825	12 (39)	115 (39)	0.942
-Asthma	4 (9)	21 (14)	0.37	2 (2)	110 (14)	0.002	0 (0)	54 (18)	0.009
-COPD	5 (11)	4 (3)	0.018	14 (16)	96 (12)	0.265	9 (29)	49 (17)	0.091
Hepatopathy	2 (5)	12 (8)	0.419	24 (28)	80 (10)	<0.001	9 (29)	28 (10)	0.001
CKD	14 (32)	12 (8)	<0.001	29 (33)	116 (14)	<0.001	10 (32)	29 (10)	<0.001
Stroke	4 (9)	6 (4)	0.191	14 (16)	56 (7)	0.003	5 (16)	14 (5)	0.011
Cancer	7 (16)	10 (7)	0.063	18 (21)	75 (9)	0.001	7 (23)	23 (8)	0.007
Immunodeficiency	3 (7)	8 (5)	0.731	9 (10)	47 (6)	0.101	4 (13)	21 (7)	0.258
Symptoms at ED									
Symptom duration before ED—days	4 (2–10)	7 (3–11)	0.047	2 (1–3)	3 (2–9)	<0.001	3 (2–3)	5 (2–10)	0.001
Cough	25 (57)	101 (69)	0.144	40 (46)	425 (53)	0.218	16 (52)	226 (77)	0.002
Dyspnea	21 (48)	60 (41)	0.416	45 (52)	393 (49)	0.622	20 (65)	165 (57)	0.391
Vital signs at ED									
Systolic BP—mmHg	133 (111–149)	135 (123–148)	0.288	123 (106–151)	138 (122–156)	<0.001	128 (106–154)	140 (124–155)	0.074
Diastolic BP—mmHg	76 (63–90)	83 (74–89)	0.037	72 (63–82)	81 (73–90)	<0.001	77 (65–85)	82 (73–89)	0.02
Heart rate—/min	91 (82–110)	88 (80–102)	0.293	100 (81–118)	87 (74–101)	<0.001	110 (77–125)	89 (76–103)	0.018
Blood oxygen saturation—%	93 (89–96)	97 (95–98)	<0.001	96 (93–99)	97 (96–98)	0.015	94 (90–96)	97 (95–98)	<0.001
Respiratory rate—/min	24 (20–28)	19 (16–23)	<0.001	21 (16–25)	18 (16–22)	0.001	25 (21–30)	19 (16–22)	<0.001
Temperature—°C	37.1 (36.5–38.3)	37.1 (36.8–38)	0.68	37.2 (36.9–38.2)	37 (36.5–37.6)	0.007	37.35 (36.9–38.8)	37 (36.6–37.5)	0.044
Laboratory parameters at ED									
Leukocytes—G/L	7.63 (5.79–10.53)	6.04 (4.65–7.55)	0.002	11.70 (7.77–14.92)	8.67 (6.79–11.21)	<0.001	12.535 (8.52–16.53)	8.87 (7.09–11.185)	0.001
Lymphocytes—%	11.4 (6.5–19.4)	21.2 (13.7–28.5)	<0.001	9.5 (5.4–14.4)	18.4 (10.5–27.3)	<0.001	7.6 (5.1–12.9)	20.1 (11.1–28)	<0.001
Lymphocytes absolute—G/L	0.77 (0.57–1.31)	1.14 (0.84–1.65)	<0.001	1.06 (0.79–1.39)	1.53 (0.93–2.14)	<0.001	1.03 (0.67–1.36)	1.67 (1.06–2.22)	<0.001
Thrombocytes—G/L	208 (146–274)	219 (180–279)	0.193	223 (178–316)	241 (197–287)	0.428	231.5 (186–313)	252 (208–291)	0.556
CRP—mg/dL	112.6 (47.6–162.7)	15.6 (1.6–46.7)	<0.001	40.1 (10.8–113.8)	6.4 (1.1–40.6)	<0.001	86.1 (34.7–129.9)	6.1 (1.3–35.4)	<0.001
Ferritin—µg/L	1206 (441–2097)	306 (132–612)	<0.001	263 (119–515)	159 (84–308)	0.001	212 (104–283)	159 (85–300)	0.432
eGFR—mL/min/1.73 m²	67 (41–98)	95 (77–105)	<0.001	72 (44–88)	91 (72–107)	<0.001	70 (48–91)	93.5 (75–110)	0.001
Sodium—mmol/L	136 (134–139)	137 (134–140)	0.31	138 (135–142)	139 (136–141)	0.772	136 (133–139)	139 (136–141)	0.057
Potassium—mmol/L	4.1 (3.7–4.6)	3.9 (3.7–4.1)	0.181	4 (3.7–4.5)	4 (3.8–4.3)	0.284	4.2 (3.6–4.5)	4 (3.8–4.2)	0.299
LDH—U/L	420 (299–531)	236 (193–300)	<0.001	295 (236–381)	209 (184–247)	<0.001	315 (237–364)	207 (184–242)	<0.001
ASAT—U/L	45 (36–64)	28 (22–40)	<0.001	33 (24–56)	26 (21–32)	<0.001	34 (22–50)	26 (21–32)	0.079
Albumin—g/L	29 (26–33)	35 (31–38)	<0.001	31 (25.5–35)	37 (33–40)	<0.001	29 (25–34)	38 (33–40)	<0.001
Hs-cTnT—ng/L	18 (9–40)	6 (4–12)	<0.001	34 (20–83)	8 (4–18)	<0.001	30 (21–51)	6 (4–15)	<0.001
NT-proBNP—pg/mL	350 (82–1909)	63 (49–145)	<0.001	945 (286–4577)	96 (49–351)	<0.001	2287 (446–11052)	81 (49–208)	<0.001

^1^ *p*-values for comparison of clinical characteristics regarding the primary composite outcome, continuous variables were compared using the Mann-Whitney-U test, and categorical variables using the Pearson χ2 test or Fisher’s exact test, as appropriate; Values are numbers (percentages) or median (interquartile range); COVID-19 = coronavirus disease 2019, COPD = chronic obstructive pulmonary disease, CKD = chronic kidney disease, ED = emergency department, BP = blood pressure, CRP = c-reactive protein, eGFR = estimated glomerular filtration rate, LDH = lactate dehydrogenase, ASAT = aspartate aminotransferase, hs-cTnT = high-sensitivity troponin T, NT-proBNP = N-terminal prohormone B-type natriuretic peptide.

**Table 4:** Predictive value of clinical parameters at ED presentation for incidence of ICU admission or death at 30 days in COVID-19 and controls.

Measures	COVID-19	Overall Controls	Respiratory Controls
	HR (95%CI)	HR (95%CI)	HR (95%CI)
Demographics			
Age in decades	1.42 (1.18–1.71)	1.35 (1.20–1.52)	1.35 (1.11–1.66)
Female	0.57 (0.30–1.08)	0.88 (0.57–1.34)	0.79 (0.39–1.63)
Comorbidities			
Cardiac disease	3.58 (1.97–6.52)	4.00 (2.60–6.15)	5.67 (2.67–12.05)
Coronary artery disease	3.20 (1.62–6.35)	2.75 (1.74–4.33)	1.99 (0.86–4.61)
Atrial fibrillation	3.74 (1.58–8.86)	2.76 (1.68–4.55)	5.28 (2.53–11.03)
Hypertension	2.30 (1.25–4.22)	2.81 (1.81–4.37)	2.76 (1.30–5.85)
Overweight	2.70 (1.47–4.96)	1.44 (0.94–2.22)	1.64 (0.79–3.37)
Diabetes	2.09 (1.11–3.94)	2.03 (1.27–3.25)	2.52 (1.19–5.34)
Ever smoker	1.49 (0.81–2.73)	1.26 (0.83–1.92)	1.66 (0.81–3.42)
-Packyears > 20y	2.34 (1.09–5.04)	1.83 (1.15–2.91)	2.27 (1.11–4.64)
COPD	2.86 (1.13–7.25)	1.38 (0.78–2.44)	1.89 (0.87–4.11)
CKD	3.37 (1.79–6.37)	2.69 (1.72–4.21)	3.78 (1.78–8.02)
Cancer	2.04 (0.91–4.59)	2.27 (1.35–3.82)	3.03 (1.31–7.04)
Immunodeficiency	1.18 (0.37, 3.82)	1.76 (0.88, 3.50)	1.81 (0.63, 5.17)
Symptoms at ED			
Symptom duration before ED—days	0.97 (0.92–1.02)	0.84 (0.77–0.92)	0.78 (0.66–0.93)
Cough	0.65 (0.36–1.18)	0.77 (0.51–1.17)	0.34 (0.17–0.68)
Dyspnea	1.26 (0.70–2.27)	1.12 (0.73–1.70)	1.38 (0.66–2.87)
Vital signs at ED – per unit increase			
Systolic BP	0.99 (0.97–1.01)	0.98 (0.97–0.99)	0.98 (0.96–0.99)
Diastolic BP	0.97 (0.94–0.99)	0.97 (0.96–0.98)	0.98 (0.96–0.99)
Heart rate	1.01 (0.99–1.03)	1.03 (1.02–1.04)	1.03 (1.01–1.05)
Blood oxygen saturation	0.92 (0.90–0.95)	0.92 (0.89–0.95)	0.85 (0.80–0.89)
Respiratory rate	1.11 (1.05–1.17)	1.09 (1.05–1.12)	1.14 (1.09–1.19)
Temperature	0.93 (0.62–1.40)	1.39 (1.11–1.74)	1.76 (1.20–2.58)
Laboratory parameters—per decades			
Leukocytes	1.534 (1.158–2.031)	2.434 (1.759–3.369)	2.708 (1.604–4.572)
Lymphocytes	0.523 (0.363–0.752)	0.465 (0.359–0.603)	0.299 (0.177–0.508)
Lymphocytes absolute	1.259 (0.707–2.242)	0.017 (0.001–0.287)	0.002 (0.000–0.317)
Thrombocytes	0.984 (0.950–1.020)	1.000 (0.976–1.024)	0.980 (0.936–1.027)
CRP	1.105 (1.072–1.140)	1.065 (1.042–1.088)	1.094 (1.060–1.129)
Ferritin	1.001 (1.001–1.002)	1.004 (1.001–1.007)	1.006 (0.998–1.014)
eGFR	0.809 (0.735–0.890)	0.842 (0.785–0.902)	0.865 (0.763–0.980)
LDH	1.035 (1.025–1.045)	1.011 (1.009–1.014)	1.009 (1.006–1.013)
ASAT	1.025 (1.010–1.039)	1.024 (1.016–1.033)	1.076 (1.034–1.119)
Hs-cTnT	1.110 (1.064–1.157)	1.005 (1.004–1.007)	1.004 (1.002–1.007)
NT-proBNP	1.001 (1.000–1.002)	1.000 (1.000–1.001)	1.000 (1.000–1.001)

ED = emergency department, ICU = intensive care unit, COVID-19 = coronavirus disease 2019, HR = hazard ratio, CI = confidence interval, COPD = chronic obstructive pulmonary disease, CKD = chronic kidney disease, BP = blood pressure, CRP = c-reactive protein, eGFR = estimated glomerular filtration rate, LDH = lactate dehydrogenase, ASAT = aspartate aminotransferase, hs-cTnT = high-sensitivity troponin T, NT-proBNP = N-terminal prohormone B-type natriuretic peptide.

**Table 5:** COLT-58-Score.

Letter	Risk Factor		Score
C	CRP	>50 mg/dL	+1
O	Oxygen saturation	91–96%	+1
		<91%	+2
L	LDH	>275 U/L	+1
T	High-sensitivity Troponin T	>14 ng/L	+1
58	Age	>58 years	+1

C, represents CRP, O, represents Oxygen saturation, L, represents LDH, T, represents high-sensitivity Troponin T, 58 represents age >58 years; CRP = c-reactive protein, LDH = lactate dehydrogenase.

**Table 6:** Performance metrics of the COLT-58-Score to rule out and rule in endpoints at different cut-off values in COVID-19.

Cut-off Value	No of Patients (%)	Sensitivity ^1^ -% (95% CI)	NPV ^1^ -% (95% CI)	Specificity ^1^ -% (95% CI)	PPV ^1^ -% (95% CI)	Composite Outcome -%	Death at 30d -%
Rule-out							
0	54 (28.3)	100 (92–100)	100 (93.4–100)	36.7 (29.4–44.8)	32.1 (24.9–40.3)	0	0
≤1	82 (42.9)	95.5 (84.9–98.7)	97.6 (91.5–99.3)	54.4 (46.4–62.3)	38.5 (29.9–47.9)	2.4	0
≤2	111 (58.1)	88.6 (76–95)	95.5 (89.9–98.1)	72.1 (64.4–78.7)	48.8 (38.1–59.5)	4.5	0
≤3	142 (74.3)	70.5 (55.8–81.8)	90.8 (85–94.6)	87.8 (81.5–92.1)	63.3 (49.3–75.3)	9.2	0
Rule-in							
≥3	80 (41.9)	88.6 (76–95)	95.5 (89.9–98.1)	72.1 (64.4–78.7)	48.8 (38.1–59.5)	48.8	16.3
≥4	49 (25.7)	70.5 (55.8–81.8)	90.8 (85–94.6)	87.8 (81.5–92.1)	63.3 (49.3–75.3)	63.3	26.5
≥5	22 (11.5)	38.6 (25.7–53.4)	84.0 (77.8–88.8)	96.6 (92.3–98.5)	77.3 (56.6–89.9)	77.3	40.9
6	8 (4.2)	15.9 (7.9–29.4)	79.8 (73.4–85)	99.3 (96.2–99.9)	87.5 (52.9–97.8)	87.5	50.0

^1^ Numbers refer to the primary composite outcome of ICU admission or death at 30 days; ICU = intensive care unit, CI = confidence interval, NPV = negative predictive value, PPV = positive predictive value.

## Data Availability

The data underlying this article will be shared on reasonable request to the corresponding author and after individual approval of the responsible ethics committee.

## Supplementary Materials

The following are available online at https://www.mdpi.com/2077-0383/10/12/2672/s1, Figure S1: Flow chart of inclusion, Figure S2: Event curve for death at 30 days in COVID-19 and controls, Figure S3: Distribution and event rate for respective blood oxygen levels in patients with any respiratory infection, Figure S4: Predictive performance of the COLT-58-Score and its components in controls, Figure S5: Predictive performance comparison of the COLT-58-Score and the CURB-65-Score in COVID-19 and controls, Table S1: Strobe statement, Table S2: Missing values, Table S3: Baseline characteristics for inpatients in COVID-19 and controls, Table S4: Comparison of the COLT-58-Score and the CURB-65-Score.

## References

[1] Vinay R., Baumann H., Biller-Andorno N. (2021). Ethics of ICU Triage during COVID-19. Br. Med. Bull..

[2] Stang A., Stang M., Jöckel K.-H. (2020). Estimated Use of Intensive Care Beds Due to COVID-19 in Germany Over Time. Dtsch. Ärztebl. Int..

[3] Swiss Academy of Medical Sciences (2020). COVID-19 Pandemic: Triage for Intensive-Care Treatment under Resource Scarcity. Swiss Med. Wkly..

[4] Guan W., Ni Z., Hu Y., Liang W., Ou C., He J., Liu L., Shan H., Lei C., Hui D.S.C. (2020). Clinical Characteristics of Coronavirus Disease 2019 in China. N. Engl. J. Med..

[5] Cummings M.J., Baldwin M.R., Abrams D., Jacobson S.D., Meyer B.J., Balough E.M., Aaron J.G., Claassen J., Rabbani L.E., Hastie J. (2020). Epidemiology, Clinical Course, and Outcomes of Critically Ill Adults with COVID-19 in New York City: A Prospective Cohort Study. Lancet.

[6] Richardson S., Hirsch J.S., Narasimhan M., Crawford J.M., McGinn T., Davidson K.W., Barnaby D.P., Becker L.B., Chelico J.D., Cohen S.L. (2020). Presenting Characteristics, Comorbidities, and Outcomes Among 5700 Patients Hospitalized With COVID-19 in the New York City Area. JAMA.

[7] Grasselli G., Zangrillo A., Zanella A., Antonelli M., Cabrini L., Castelli A., Cereda D., Coluccello A., Foti G., Fumagalli R. (2020). Baseline Characteristics and Outcomes of 1591 Patients Infected With SARS-CoV-2 Admitted to ICUs of the Lombardy Region, Italy. JAMA.

[8] Wiersinga W.J., Rhodes A., Cheng A.C., Peacock S.J., Prescott H.C. (2020). Pathophysiology, Transmission, Diagnosis, and Treatment of Coronavirus Disease 2019 (COVID-19): A Review. JAMA.

[9] Huang C., Wang Y., Li X., Ren L., Zhao J., Hu Y., Zhang L., Fan G., Xu J., Gu X. (2020). Clinical Features of Patients Infected with 2019 Novel Coronavirus in Wuhan, China. Lancet.

[10] Zhou F., Yu T., Du R., Fan G., Liu Y., Liu Z., Xiang J., Wang Y., Song B., Gu X. (2020). Clinical Course and Risk Factors for Mortality of Adult Inpatients with COVID-19 in Wuhan, China: A Retrospective Cohort Study. Lancet.

[11] Wu Z., McGoogan J.M. (2020). Characteristics of and Important Lessons From the Coronavirus Disease 2019 (COVID-19) Outbreak in China: Summary of a Report of 72 314 Cases From the Chinese Center for Disease Control and Prevention. JAMA.

[12] Zhang X., Lewis A.M., Moley J.R., Brestoff J.R. (2021). A Systematic Review and Meta-Analysis of Obesity and COVID-19 Outcomes. Sci. Rep..

[13] Yang J., Hu J., Zhu C. (2021). Obesity Aggravates COVID-19: A Systematic Review and Meta-Analysis. J. Med. Virol..

[14] Herold T., Jurinovic V., Arnreich C., Lipworth B.J., Hellmuth J.C., Weinberger T. (2020). Elevated Levels of IL-6 and CRP Predict the Need for Mechanical Ventilation in COVID-19. J. Allergy Clin. Immunol..

[15] Liu F., Li L., Xu M., Wu J., Luo D. (2020). Prognostic Value of Interleukin-6, C-Reactive Protein, and Procalcitonin in Patients with COVID-19. J. Clin. Virol..

[16] Grasselli G., Greco M., Zanella A., Albano G., Antonelli M., Bellani G., Bonanomi E., Cabrini L., Carlesso E., Castelli G. (2020). Risk Factors Associated With Mortality Among Patients With COVID-19 in Intensive Care Units in Lombardy, Italy. JAMA Intern. Med..

[17] Ji D., Zhang D., Xu J., Chen Z., Yang T., Zhao P., Chen G., Cheng G., Wang Y., Bi J. (2020). Prediction for Progression Risk in Patients With COVID-19 Pneumonia: The CALL Score. Clin. Infect. Dis..

[18] Petrakis D., Margină D., Tsarouhas K., Tekos F., Stan M., Nikitovic D., Kouretas D., Spandidos D.A., Tsatsakis A. (2020). Obesity —A Risk Factor for Increased COVID-19 Prevalence, Severity and Lethality (Review). Mol. Med. Rep..

[19] Karagiannidis C., Mostert C., Hentschker C., Voshaar T., Malzahn J., Schillinger G., Klauber J., Janssens U., Marx G., Weber-Carstens S. (2020). Case Characteristics, Resource Use, and Outcomes of 10 021 Patients with COVID-19 Admitted to 920 German Hospitals: An Observational Study. Lancet Respir. Med..

[20] Higuchi T., Nishida T., Iwahashi H., Morimura O., Otani Y., Okauchi Y., Yokoe M., Suzuki N., Inada M., Abe K. (2021). Early Clinical Factors Predicting the Development of Critical Disease in Japanese Patients with COVID-19: A Single-Center, Retrospective, Observational Study. J. Med. Virol..

[21] Ludwig M., Jacob J., Basedow F., Andersohn F., Walker J. (2021). Clinical Outcomes and Characteristics of Patients Hospitalized for Influenza or COVID-19 in Germany. Int. J. Infect. Dis..

[22] Piroth L., Cottenet J., Mariet A.-S., Bonniaud P., Blot M., Tubert-Bitter P., Quantin C. (2021). Comparison of the Characteristics, Morbidity, and Mortality of COVID-19 and Seasonal Influenza: A Nationwide, Population-Based Retrospective Cohort Study. Lancet Respir. Med..

[23] Brehm T.T., van der Meirschen M., Hennigs A., Roedl K., Jarczak D., Wichmann D., Frings D., Nierhaus A., Oqueka T., Fiedler W. (2021). Comparison of Clinical Characteristics and Disease Outcome of COVID-19 and Seasonal Influenza. Sci. Rep..

[24] Lim W.S., van der Eerden M.M., Laing R., Boersma W.G., Karalus N., Town G.I., Lewis S.A., Macfarlane J.T. (2003). Defining Community Acquired Pneumonia Severity on Presentation to Hospital: An International Derivation and Validation Study. Thorax.

[25] Von Elm E., Altman D.G., Egger M., Pocock S.J., Gøtzsche P.C., Vandenbroucke J.P. (2014). The Strengthening the Reporting of Observational Studies in Epidemiology (STROBE) Statement: Guidelines for Reporting Observational Studies. Int. J. Surg..

[26] Rubin D.B. (1987). Multiple Imputation for Nonresponse in Surveys.

[27] Pencina M.J., Agostino R.B.D., Agostino R.B.D., Vasan R.S. (2008). Evaluating the Added Predictive Ability of a New Marker: From Area under the ROC Curve to Reclassification and Beyond. Stat. Med..

[28] Pencina M.J., Steyerberg E.W., D’Agostino R.B. (2011). Extensions of Net Reclassification Improvement Calculations to Measure Usefulness of New Biomarkers. Stat. Med..

[29] Metlay J.P., Waterer G.W., Long A.C., Anzueto A., Brozek J., Crothers K., Cooley L.A., Dean N.C., Fine M.J., Flanders S.A. (2019). Diagnosis and Treatment of Adults with Community-Acquired Pneumonia. An Official Clinical Practice Guideline of the American Thoracic Society and Infectious Diseases Society of America. Am. J. Respir. Crit. Care Med..

[30] Ebell M.H. (2006). Outpatient vs. Inpatient Treatment of Community-Acquired Pneumonia. Fam. Pract. Manag..

[31] De Jong A., Molinari N., Pouzeratte Y., Verzilli D., Chanques G., Jung B., Futier E., Perrigault P.-F., Colson P., Capdevila X. (2015). Difficult Intubation in Obese Patients: Incidence, Risk Factors, and Complications in the Operating Theatre and in Intensive Care Units. BJA Br. J. Anaesth..

[32] Capoferri G., Osthoff M., Egli A., Stoeckle M., Bassetti S. (2021). Relative Bradycardia in Patients with COVID-19. Clin. Microbiol. Infect..

[33] Ikeuchi K., Saito M., Yamamoto S., Nagai H., Adachi E. (2020). Relative Bradycardia in Patients with Mild-to-Moderate Coronavirus Disease, Japan. Emerg. Infect. Dis..

[34] Chen R., Liang W., Jiang M., Guan W., Zhan C., Wang T., Tang C., Sang L., Liu J., Ni Z. (2020). Risk Factors of Fatal Outcome in Hospitalized Subjects With Coronavirus Disease 2019 From a Nationwide Analysis in China. Chest.

[35] Horby P., Lim W.S., Emberson K.R., Mafham M., Bell J.L., Linsell L., Staplin N., Brightling C., Ustianowski A., The RECOVERY Collaborative Group Dexamethasone in Hospitalized Patients with Covid-19—Preliminary Report. N. Engl. J. Med..

[36] Pan H., Peto R., Henao-Restrepo A.-M., Preziosi M.-P., Sathiyamoorthy V., Abdool Karim Q., Alejandria M.M., Hernández García C., Kieny M.-P., WHO Solidarity Trial Consortium (2021). Repurposed Antiviral Drugs for Covid-19—Interim WHO Solidarity Trial Results. N. Engl. J. Med..

[37] Tworek A., Jaroń K., Uszyńska-Kałuża B., Rydzewski A., Gil R., Deptała A., Franek E., Wójtowicz R., Życińska K., Walecka I. (2021). Convalescent Plasma Treatment Is Associated with Lower Mortality and Better Outcomes in High-Risk COVID-19 Patients—Propensity-Score Matched Case-Control Study. Int. J. Infect. Dis..

