# Supplementary material for: Direct Comparison of Clinical Characteristics, Outcomes, and Risk Prediction in Patients with COVID-19 and Controls—A Prospective Cohort Study
Source: J Clin Med. 2021 Jun 17;10(12):2672. doi: 10.3390/jcm10122672 (PMC8234319; doi:10.3390/jcm10122672)
Supplement: Supplementary file 1 [file jcm-10-02672-s001.zip › jcm-1238746-supplementary.pdf]

Supplementary

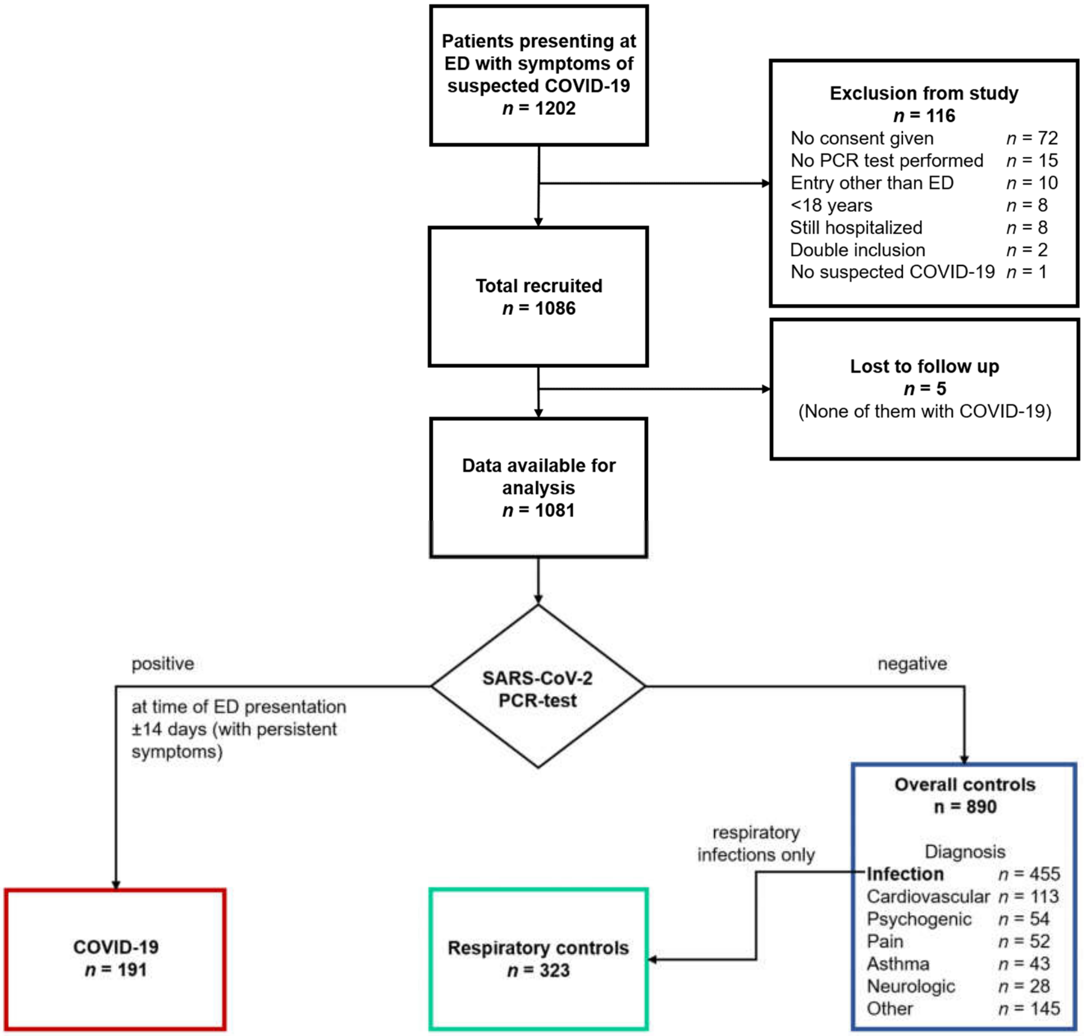

Figure S1. Flow chart of inclusion. ED = emergency department, COVID-19 = coronavirus disease 2019, SARS-CoV-2 = severe acute respiratory syndrome - coronavirus 2, PCR = polymerase chain reaction.

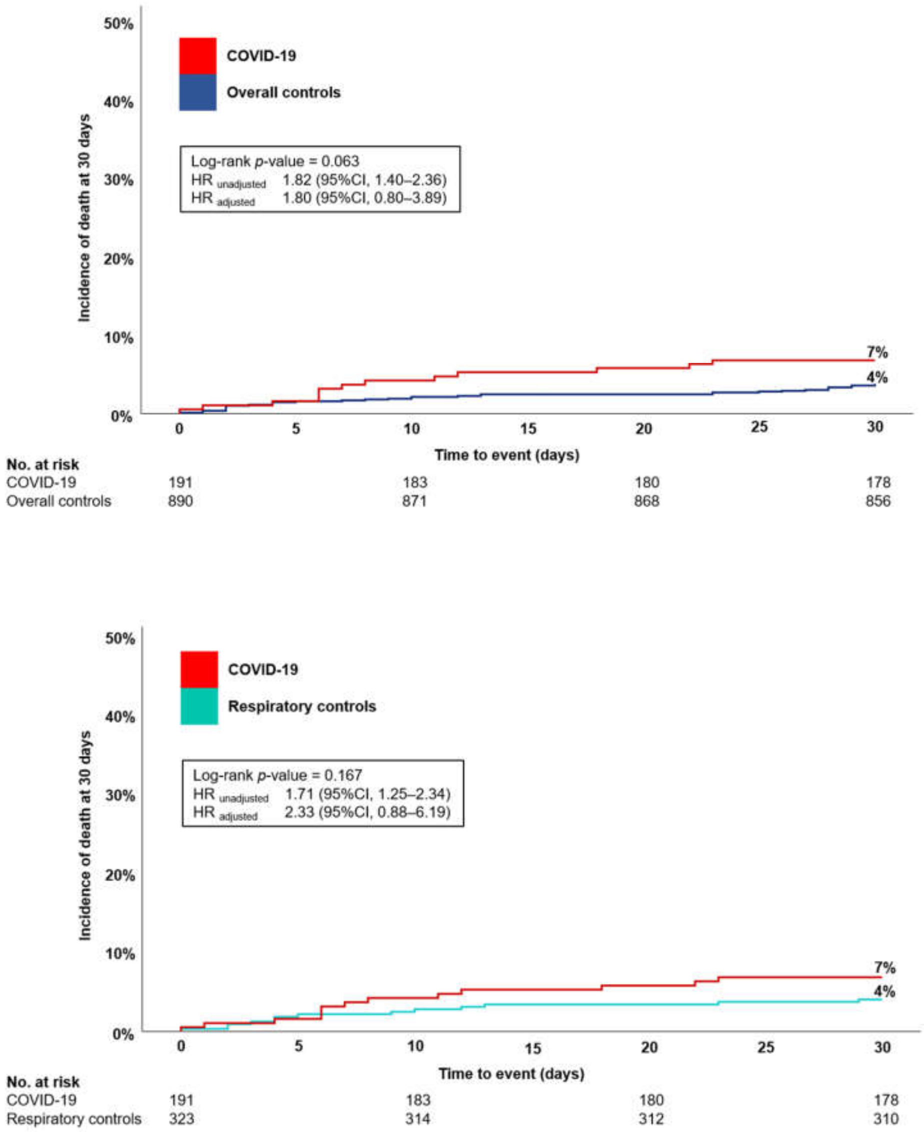

Figure S2. Event curve for death at 30 days in COVID-19 and controls. First panel shows incidence of death at 30 days for COVID-19 vs. overall controls; Second panel shows incidence of death at 30 days for COVID-19 vs. respiratory controls, adjustment was made for cardiac disease, pneumopathy, overweight, diabetes, active smoking, CRP, and blood oxygen saturation; COVID-19 = coronavirus disease 2019, HR = hazard ratio, CI = confidence interval, CRP = c-reactive protein.

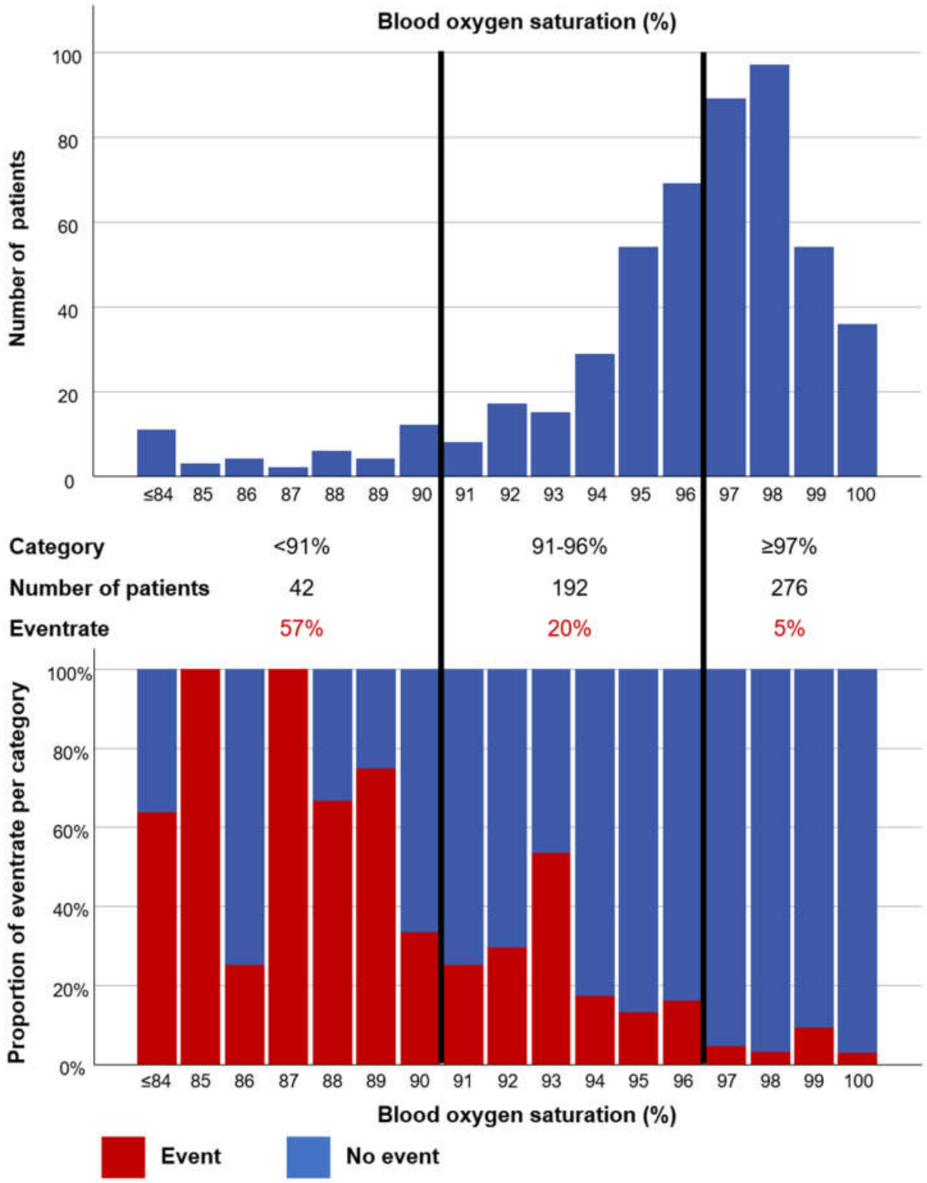

Figure S3. Distribution and event rate for respective blood oxygen levels in patients with any respiratory infection (n = 514, blood oxygen saturation available in n = 510, missing in n = 4). Upper panel shows the distribution of blood oxygen saturation levels in patients with any respiratory infection; Lower panel shows the event rate of the respective blood oxygen saturation levels.

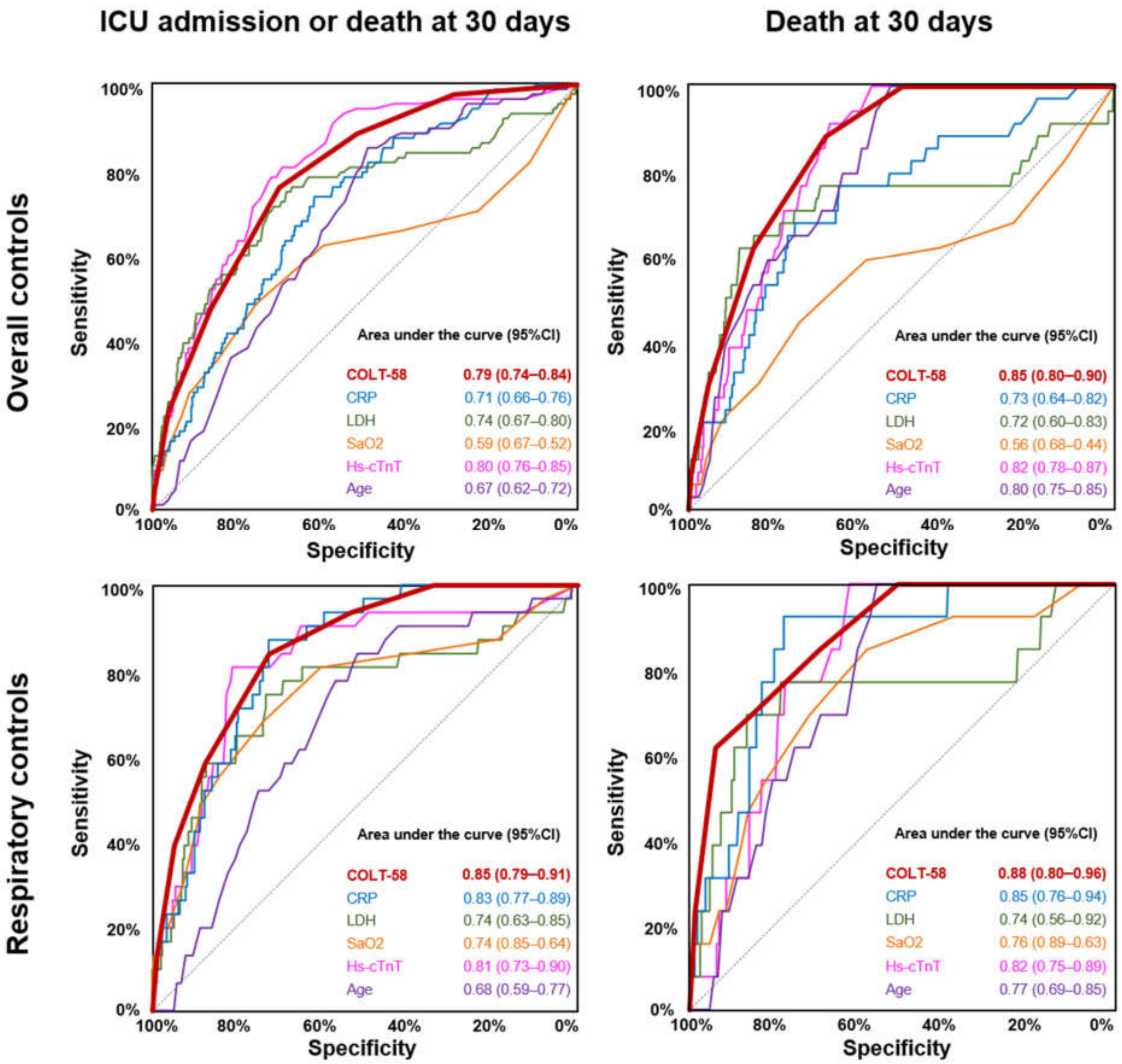

Figure S4. Predictive performance of the COLT-58-Score and its components in controls. Upper left panel shows the AUROC for ICU admission and death at 30 days for the COLT-58-Score, CRP, LDH, SaO2, hs-cTnT, and age in overall controls; Upper right panel shows the AUROC for death at 30 days for the COLT-58-Score, CRP, LDH, SaO2, hs-cTnT, and age in overall controls; Lower left panel shows the AUROC for ICU admission and death at 30 days for the COLT-58-Score, CRP, LDH, SaO2, hs-cTnT, and age in respiratory controls. Lower right panel shows the AUROC for death at 30 days for the COLT-58-Score, CRP, LDH, SaO2, hs-cTnT, and age in respiratory controls; AUROC = area under the receiver operating characteristic curve, ICU = intensive care unit, CI = confidence interval, CRP = c-reactive protein, LDH = lactate dehydrogenase, SaO2 = blood oxygen saturation, hs-cTnT = high-sensitivity troponin T.

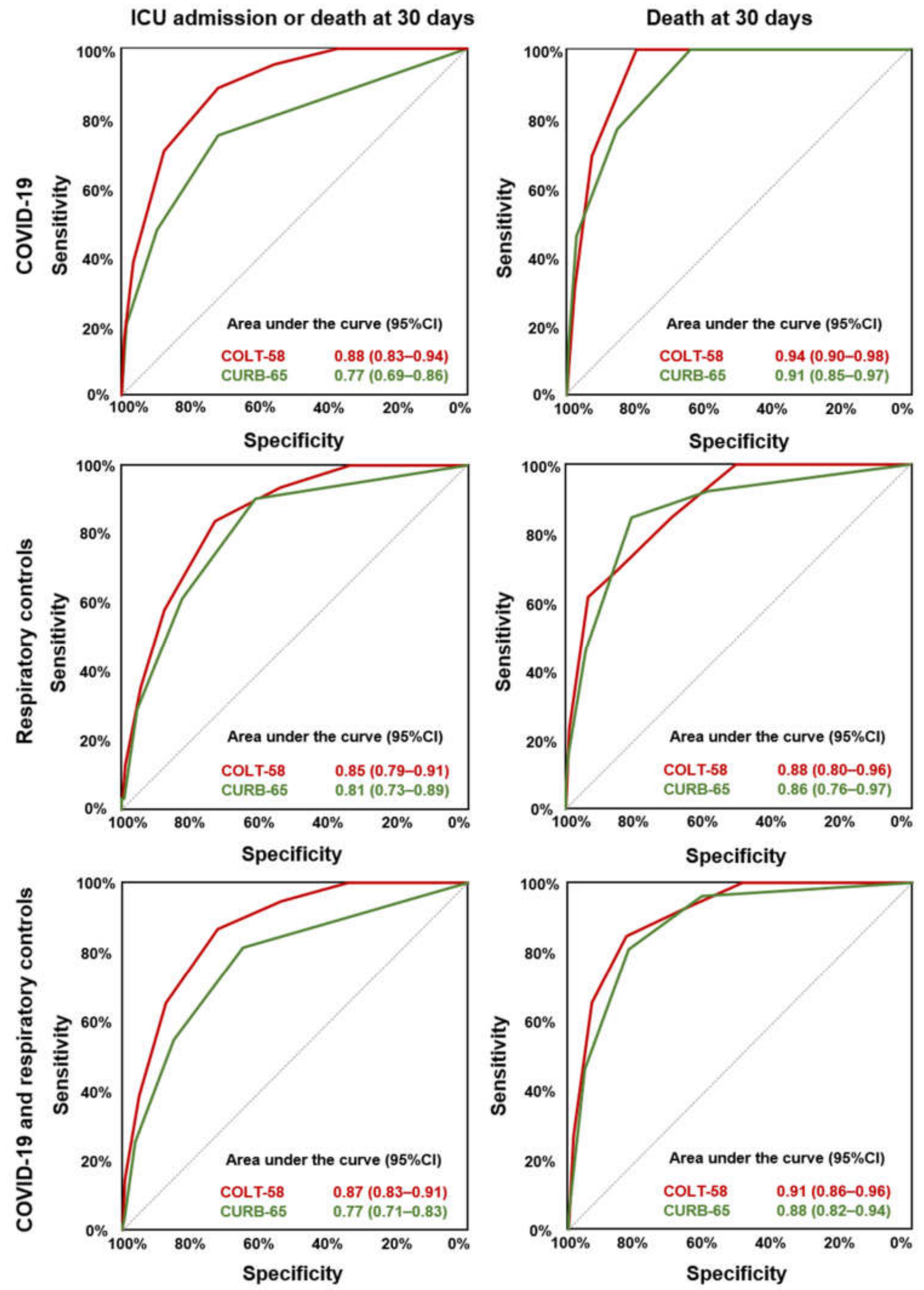

Figure S5. Predictive performance comparison of the COLT-58-Score and the CURB-65-Score in COVID-19 and controls. Upper left panel shows the AUROC for the primary composite outcome of ICU admission and death at 30 days for the COLT-58-Score and the CURB-65-Score in COVID-19; Upper right panel shows the AUROC for death at 30 days for the COLT-58-Score and the CURB-65-Score in COVID-19; Middle left panel shows the AUROC for the primary composite outcome of ICU admission and death at 30 days for the COLT-58-Score and the CURB-65-Score in respiratory controls; Middle right panel shows the AUROC for death at 30 days for the COLT-58-Score and the CURB-65-Score in respiratory controls; Lower left panel shows the AUROC for the primary composite outcome of ICU admission and death at 30 days for the COLT-58-Score and the CURB-65-Score in patients with any respiratory infection; Lower right panel shows the AUROC for death at 30 days for the COLT-58-Score and the CURB-65-Score in patients with any respiratory infection; COVID-19 = coronavirus disease 2019, AUROC = area under the receiver operating characteristic curve, ICU = intensive care unit, CI = confidence interval.

Table S1. Strobe statement.

	Item No	Recommendation	Page No
Title and abstract	1	(a) Indicate the study's design with a commonly used term in the title or the abstract	1
		(b) Provide in the abstract an informative and balanced summary of what was done and what was found	1
Introduction			
Background/rationale	2	Explain the scientific background and rationale for the investigation being reported	2
Objectives	3	State specific objectives, including any prespecified hypotheses	2
Methods			
Study design	4	Present key elements of study design early in the paper	2–3
Setting	5	Describe the setting, locations, and relevant dates, including periods of recruitment, exposure, follow-up, and data collection	2–3
Participants	6	(a) Give the eligibility criteria, and the sources and methods of selection of participants. Describe methods of follow-up	2–3
		(b) For matched studies, give matching criteria and number of exposed and unexposed	-
Variables	7	Clearly define all outcomes, exposures, predictors, potential confounders, and effect modifiers. Give diagnostic criteria, if applicable	3
Data sources/ measurement	8	For each variable of interest, give sources of data and details of methods of assessment (measurement). Describe comparability of assessment methods if there is more than one group	2–3
Bias	9	Describe any efforts to address potential sources of bias	17
Study size	10	Explain how the study size was arrived at	4
Quantitative variables	11	Explain how quantitative variables were handled in the analyses. If applicable, describe which groupings were chosen and why	3–4
Statistical methods	12	(a) Describe all statistical methods, including those used to control for confounding	3–4
		(b) Describe any methods used to examine subgroups and interactions	4
		(c) Explain how missing data were addressed	4
		(d) If applicable, explain how loss to follow-up was addressed	4
		(e) Describe any sensitivity analyses	3–4
Results			
Participants	13	(a) Report numbers of individuals at each stage of study—eg numbers potentially eligible, examined for eligibility, confirmed eligible, included in the study, completing follow-up, and analysed	4
		(b) Give reasons for non-participation at each stage	21
		(c) Consider use of a flow diagram	Figure S1
Descriptive data	14	(a) Give characteristics of study participants (eg demographic, clinical, social) and information on exposures and potential confounders	5–6, Table 1
		(b) Indicate number of participants with missing data for each variable of interest	Table S2
		(c) Summarise follow-up time (eg, average and total amount)	6–8
Outcome data	15	Report numbers of outcome events or summary measures over time	7
Main results	16	(a) Give unadjusted estimates and, if applicable, confounder-adjusted estimates and their precision (eg, 95% confidence interval). Make clear which confounders were adjusted for and why they were included	3, 6–8
		(b) Report category boundaries when continuous variables were categorized	7
		(c) If relevant, consider translating estimates of relative risk into absolute risk for a meaningful time period	-
Other analyses	17	Report other analyses done—eg analyses of subgroups and interactions, and sensitivity analyses	8–17
Discussion			
Key results	18	Summarise key results with reference to study objectives	17–19
Limitations	19	Discuss limitations of the study, taking into account sources of potential bias or imprecision. Discuss both direction and magnitude of any potential bias	19
Interpretation	20	Give a cautious overall interpretation of results considering objectives, limitations, multiplicity of analyses, results from similar studies, and other relevant evidence	17–19
Generalisability	21	Discuss the generalisability (external validity) of the study results	17–19
Other information			
Funding	22	Give the source of funding and the role of the funders for the present study and, if applicable, for the original study on which the present article is based	20

Table S2. Missing values.

Measures	Missing Values Study Population *n* = 1081		COVID-19 *n* = 191		Overall Controls *n* = 890		Respiratory Controls *n* = 323	
	N	%	N	%	N	%	N	%
Age	0	0.0%	0	0.0%	0	0.0%	0	0.0%
Systolic BP	79	7.3%	19	9.9%	60	6.7%	25	2.3%
Diastolic BP	96	8.9%	23	12.0%	73	8.2%	29	2.7%
Heart rate	59	5.5%	16	8.4%	43	4.8%	15	1.4%
Blood oxygen saturation	7	0.6%	2	1.0%	5	0.6%	2	0.2%
Respiratory rate	74	6.8%	18	9.4%	56	6.3%	16	1.5%
Temperature	72	6.7%	22	11.5%	50	5.6%	18	1.7%
Leukocytes	13	1.2%	0	0.0%	13	1.5%	6	0.6%
Lymphocytes	31	2.9%	7	3.7%	24	2.7%	11	1.0%
Lymphocytes absolute	31	2.9%	7	3.7%	24	2.7%	11	1.0%
Thrombocytes	24	2.2%	5	2.6%	19	2.1%	9	0.8%
CRP	10	0.9%	1	0.5%	9	1.0%	5	0.5%
Ferritin	55	5.1%	7	3.7%	48	5.4%	16	1.5%
eGFR	10	0.9%	7	3.7%	15	1.7%	7	0.6%
Sodium	22	2.0%	7	3.7%	15	1.7%	7	0.6%
Potassium	22	2.0%	7	3.7%	15	1.7%	7	0.6%
LDH	58	5.4%	7	3.7%	51	5.7%	26	2.4%
ASAT	57	5.3%	8	4.2%	49	5.5%	25	2.3%
Albumin	24	2.2%	8	4.2%	16	1.8%	8	0.7%
Hs-cTnT	56	5.2%	13	6.8%	43	4.8%	15	1.4%
NT-pro-BNP	105	9.7%	27	14.1%	78	8.8%	26	2.4%

BP = blood pressure, CRP = c-reactive protein, eGFR = estimated glomerular filtration rate, LDH = lactate dehydrogenase, ASAT = aspartate aminotransferase, hs-cTnT = high-sensitivity troponin T, NT-proBNP = N-terminal prohormone B-type natriuretic peptide.

Table S3. Baseline characteristics for inpatients in COVID-19 and controls.

Measures	COVID-19 *n* = 114	Overall Controls *n* = 444	*p*-Value[1]	Respiratory Controls *n* = 138	*p*-Value[2]
Demographics					
Age—years	62 (52, 73)	71 (56, 81)	0.002	71 (56, 79)	0.01
Female	43 (38)	179 (40)	0.613	55 (40)	0.729
Comorbidities—no (%)					
Cardiac disease	31 (27)	209 (47)	<0.001	66 (48)	0.001
Valvular cardiopathy	8 (7)	46 (10)	0.282	13 (9)	0.492
Coronary artery disease	18 (16)	110 (25)	0.042	33 (24)	0.11
Prior myocardial infarction	7 (6)	61 (14)	0.027	17 (12)	0.096
Atrial fibrillation	8 (7)	78 (18)	0.005	28 (20)	0.003
Hypertension	65 (57)	240 (54)	0.571	80 (58)	0.879
Overweight	62 (54)	160 (36)	<0.001	44 (32)	<0.001
Diabetes	33 (29)	116 (26)	0.544	37 (27)	0.706
Ever smoker	38 (33)	194 (44)	0.045	85 (62)	<0.001
-Active smoker	7 (6)	104 (23)	<0.001	49 (36)	<0.001
-Packyears >20	16 (14)	110 (25)	0.014	53 (38)	<0.001
Pneumopathy	22 (19)	139 (31)	0.012	64 (46)	<0.001
-Asthma	12 (11)	28 (6)	0.119	14 (10)	0.921
-COPD	8 (7)	76 (17)	0.007	42 (30)	<0.001
Hepatopathy	9 (8)	69 (16)	0.036	24 (17)	0.026
CKD	25 (22)	129 (29)	0.129	32 (23)	0.812
Stroke	8 (7)	58 (13)	0.075	17 (12)	0.161
Cancer	13 (11)	70 (16)	0.243	23 (17)	0.235
Immunodeficiency	8 (7)	42 (9)	0.415	15 (11)	0.291
Symptoms at ED(%)					
Symptom duration before ED—days	7 (3, 10)	3 (1, 7)	<0.001	3 (2, 7)	<0.001
Cough	76 (67)	196 (44)	<0.001	91 (66)	0.904
Dyspnea	50 (44)	197 (44)	0.922	84 (61)	0.007
Vital signs at ED					
Systolic BP—mmHg	134 (120, 147)	135 (117, 157)	0.507	137 (120, 159)	0.328
Diastolic BP—mmHg	80 (70, 90)	80 (69, 87)	0.319	80 (70, 87)	0.774
Heart rate—/min	90 (80, 105)	91.5 (78, 108)	0.883	94.5 (78, 109)	0.386
Blood oxygen saturation—%	95 (93, 97)	96 (94, 98)	0.005	95 (92, 97)	0.986
Respiratory rate—/min	23 (16, 25)	21 (16, 25)	0.209	22.5 (19, 27)	0.299
Temperature—°C	37.35 (36.8, 38.2)	37.2 (36.8, 38.2)	0.425	37.2 (36.9, 38.2)	0.78
Laboratory parameters at ED					
Leukocytes—G/L	6.67 (5.13, 8.89)	10.23 (7.46, 14.07)	<0.001	10.50 (8.29, 14.09)	<0.001
Lymphocytes—%	15.3 (8.3, 21.3)	11.1 (6, 16.7)	<0.001	9.9 (5.6, 16.3)	<0.001
Lymphocytes absolute—G/L	0.96 (0.60, 1.33)	1.035 (0.70, 1.60)	0.069	1.04 (0.70, 1.60)	0.106
Thrombocytes—G/L	219 (171, 282)	234 (186, 303)	0.115	251.5 (202, 298)	0.023

CRP—mg/dL	58.6 (31.5, 126.9)	35.6 (6.9, 94.9)	<0.001	46.0 (10.2, 117.3)	0.044
Ferritin—µg/L	705 (330, 1263)	228 (114, 477)	<0.001	220 (109, 384)	<0.001
eGFR—mL/min/1.73 m^2	81 (54, 96)	76 (51, 95)	0.513	77 (56, 97)	0.755
Sodium—mmol/L	135 (133, 139)	138 (134, 141)	0.001	138 (134, 141)	0.013
Potassium—mmol/L	3.9 (3.7, 4.4)	4.1 (3.8, 4.4)	0.074	4.1 (3.8, 4.4)	0.033
LDH—U/L	333 (254, 444)	233 (196, 296)	<0.001	232 (195, 315)	<0.001
ASAT—U/L	39 (27, 53)	27 (21, 39)	<0.001	28 (22, 40)	<0.001
Albumin—g/L	31 (28, 35)	33 (29, 37)	0.003	32 (29, 36)	0.252
Hs-cTnT	13 (6, 22)	21 (9, 38)	<0.001	19 (9, 36)	0.003
NT-pro-BNP—pg/mL	114 (49, 552)	420 (129, 1624)	<0.001	323 (147, 1478)	<0.001

[1] *p*-value for comparison of COVID-19 with overall controls; [2] *p*-value for comparison of COVID-19 with respiratory controls. Continuous variables were compared using the Mann-Whitney-U test, and categorical variables using the Pearson χ2 test or Fisher's exact test, as appropriate; missing variables are listed in supplemental Table S2 Values are numbers (percentages) or median (interquartile range); COVID-19 = coronavirus disease 2019, COPD = chronic obstructive pulmonary disease, CKD = chronic kidney disease, ED = emergency department, BP = blood pressure, CRP = c-reactive protein, eGFR = estimated glomerular filtration rate, LDH = lactate dehydrogenase, ASAT = aspartate aminotransferase, hs-cTnT = high-sensitivity troponin T, NT-proBNP = N-terminal prohormone B-type natriuretic peptide.

Table S4. Comparison of the COLT-58-Score and the CURB-65-Score.

Measures	AUROC		*p*-Value	NRI (%)	*p*-Value
	COLT-58-Score	CURB-65-Score			
COVID-19 (*n* = 191)					
ICU admission or death at 30 days	0.88 (0.83–0.94)	0.77 (0.69–0.86)	<0.001	30.04	0.013
Death at 30 days	0.94 (0.90–0.98)	0.91 (0.85–0.97)	0.305	14.87	0.474
Respiratory controls (*n* = 323)					
ICU admission or death at 30 days	0.85 (0.79–0.91)	0.81 (0.73–0.89)	0.288	15.87	0.255
Death at 30 days	0.88 (0.80–0.96)	0.86 (0.76–0.97)	0.832	-3.6	0.877
Any respiratory infection (*n* = 514)					
ICU admission or death at 30 days	0.87 (0.83–0.91)	0.77 (0.71–0.83)	<0.001	26.92	0.003
Death at 30 days	0.91 (0.86–0.96)	0.88 (0.82–0.94)	0.500	7.3	0.639

AUROC = area under the receiver operating characteristic curve, NRI = net reclassification improvement, COVID-19 = coronavirus disease 2019.
